# Interpretable multi-center machine learning model driven by facial image features for non-invasive early risk assessment of lung cancer

**DOI:** 10.3389/fphys.2026.1835790

**Published:** 2026-05-15

**Authors:** Yulin Shi, Yi Chun, Shuyi Zhang, Xiaoyan Xu, Wenlian Chen, Lijing Jiao, Lixin Wang, Liping Tu, Jiatuo Xu

**Affiliations:** 1 Teaching Experiment and Training Center, Academic Affairs Office, Shanghai University of Traditional Chinese Medicine, Shanghai, China; 2 Shanghai Key Laboratory of Health Identification and Assessment, School of Traditional Chinese Medicine, Shanghai University of Traditional Chinese Medicine, Shanghai, China; 3 Longhua Hospital Shanghai University of Traditional Chinese Medicine, Shanghai, China; 4 Yueyang Hospital of Integrated Traditional Chinese and Western Medicine, Shanghai University of Traditional Chinese Medicine, Shanghai, China; 5 Shanghai Pulmonary Hospital, Shanghai, China; 6 School of Artificial Intelligence in Traditional Chinese Medicine, Shanghai University of Traditional Chinese Medicine, Shanghai, China; 7 Seventh People’s Hospital of Shanghai University of Traditional Chinese Medicine, Shanghai, China

**Keywords:** benign pulmonary nodule, facial image feature, interpretable machine learning, lung cancer, multicenter study, non-invasive diagnosis

## Abstract

**Background:**

Early identification of lung cancer is critical for improving patient survival and prognosis. Conventional pathological biopsy is invasive, and computed tomography (CT) involves ionizing radiation risks. Facial imaging, as a non-invasive and accessible biological signal, is a promising novel approach for lung cancer auxiliary diagnosis.

**Objective:**

To develop and validate a non-invasive lung cancer auxiliary diagnosis model based on facial image features, and explore the value of facial imaging in lung cancer early screening.

**Methods:**

Multi-center patients with benign pulmonary nodules and lung cancer were enrolled in this study. Facial images were collected via the TFDA-1 Digital Tongue and Face Diagnosis Instrument, with 124 features extracted. Logistic regression was used for feature selection and simple correlation analysis of facial image features, based on which four machine learning models (XGBoost, LightGBM, SVM, GBDT) were constructed. Model training and optimization were performed using 10-fold cross-validation combined with grid search for hyperparameter tuning. Model performance was comprehensively evaluated using Accuracy, Precision, Sensitivity, Specificity, F1-Score, area under the curve (AUC), average precision (AP), and Brier Score; pairwise comparisons of AUCs among models were conducted using the DeLong test. Clinical utility was assessed using calibration curves and decision curves; model interpretability was analyzed via the SHapley Additive exPlanations (SHAP) method; and generalization capability was validated using an independent external validation cohort.

**Results:**

The XGBoost model achieved the best overall performance, with an AUC of 0.900 and accuracy of 0.807 in the internal test set, and an AUC of 0.906 and accuracy of 0.813 in the external validation set, demonstrating favorable generalization stability. DeLong test showed that XGBoost achieved the highest AUC in the internal test set without significant differences among models (all *P* ≥ 0.05). In external validation, its AUC was significantly higher than GBDT, LightGBM (*P* < 0.001) and SVM (*P* < 0.05).

**Conclusion:**

We successfully constructed a non-invasive auxiliary screening model for lung cancer using facial image features. Facial imaging shows significant value in early lung cancer screening, providing a novel, accessible strategy to improve the popularization and availability of lung cancer early screening in clinical practice.

## 1 Introduction

Lung cancer is one of the most threatening malignant tumors worldwide, imposing an immense epidemiological burden on the global population. Statistically, its incidence and mortality have long ranked among the highest across all malignancies worldwide (Han et al., 2024; Siegel et al., 2025). Studies have demonstrated that the 5-year overall survival rate of lung cancer is below 20% (Sung et al., 2021), and early diagnosis combined with appropriate therapeutic interventions can effectively increase the survival rate of affected patients by 20 percentage points (Lin et al., 2019). However, the rate of early diagnosis for lung cancer remains relatively low, which results in the majority of patients being confirmed at an advanced stage; thus, early screening and diagnosis are of pivotal importance for improving patient prognosis and alleviating the global disease burden. At present, although imaging examinations such as chest computed tomography (CT) have been widely adopted in clinical screening, they are associated with inherent limitations including radiation exposure (Aberle et al., 2011; Bosch de Basea et al., 2023). As the gold standard for lung cancer diagnosis, pathological biopsy is not suitable for population-based screening due to its invasive nature (Gould et al., 2013). Therefore, the development of non-invasive, safe, convenient and highly efficient tools for the early risk warning of lung cancer has become an urgent unmet need in the current field of lung cancer prevention and treatment.

According to Inspection in Traditional Chinese Medicine (TCM), changes in facial complexion are extrinsic manifestations of visceral functions and qi-blood circulation, which can serve as a basis for the early identification of diseases (Lin and Wang, 2020; Tian et al., 2024). With the advancement of computer vision and machine learning technologies, artificial intelligence (AI) has emerged as an effective tool for lung cancer diagnosis (Huang et al., 2023; Quanyang et al., 2024). Research on auxiliary diagnosis and early warning of diseases based on facial image features has achieved breakthroughs in multiple fields, thus providing a replicable approach for lung cancer early warning. Zhang J et al (Zhang et al., 2016). proposed a fuzzy support vector machine (SVM) algorithm based on color modeling, which enables the accurate identification of facial complexions corresponding to different TCM constitutions by extracting color features such as RGB and HSV to construct a predictive model. Zhang N et al (Zhang et al., 2024)developed a multi-feature learning model that integrates multi-dimensional features including color and texture, and this model achieved a performance with an area under the curve (AUC) of 0.87 in the screening of various diseases. Shu T et al (Shu et al., 2017). focused on specific facial regions, compared the performance of texture feature extractors (e.g., gray-level co-occurrence matrix [GLCM], local binary pattern [LBP]) in diabetes detection, and found that cheek texture indices were significantly correlated with blood glucose levels.

In clinical disease applications, Jin B et al (Jin et al., 2020). proposed a deep transfer learning strategy that transfers pre-trained models from the facial recognition domain to the task of facial image-based disease diagnosis, alleviating the issue of insufficient medical data volume and thus improving the model accuracy by 10–15% compared with traditional methods. Lin S et al (Lin et al., 2020). innovatively applied deep learning techniques to the early risk warning of coronary heart disease, and the early warning model constructed based on facial photographs achieved an AUC of 0.78 for this disease. Furthermore, Fadwa A et al (Alrowais et al., 2025). integrated multi-dimensional facial metrics to develop a predictive model for acute stroke that fuses transfer learning and optimization algorithms.

Although facial image analysis technology has achieved notable achievements in the field of disease diagnosis, research on lung cancer risk warning based on facial image features remains in its infancy. The limited existing studies are mostly characterized by small-sample, single-center designs, lacking large-sample cohorts and external validation, which results in insufficient generalization ability. To address this gap, the present study focuses on core clinical demands and aims to develop a machine learning model based on multi-dimensional facial image features to realize non-invasive risk warning of lung cancer. The specific research contents are as follows: (1) Extract multi-color space features (including RGB, HSV, Lab and YCrCb) and GLCM texture features of human faces, and screen out the core predictive features associated with lung cancer risk by using the logistic regression method; (2) Construct four machine learning models, namely XGBoost, LightGBM, SVM and GBDT, and perform hyperparameter optimization via stratified 10-fold cross-validation combined with the grid search method; conduct internal and external validation of the models through comprehensive evaluation metrics such as AUC, accuracy and specificity, combined with calibration curves and decision curve analysis (DCA), so as to comprehensively evaluate the predictive performance of the models; (3) Adopt the SHapley Additive exPlanations (SHAP) method to carry out model interpretability analysis, and clarify the core facial features underpinning lung cancer risk prediction and their interaction effects. This study is expected to provide a non-invasive, convenient and interpretable auxiliary tool for lung cancer risk warning, and a cost-effective, non-invasive supplementary screening strategy for individuals at risk of lung cancer. Thereby, it will improve the accessibility of early lung cancer screening and offer a novel technical approach for the early detection and early intervention of lung cancer.

## 2 Dataset and preprocessing

### 2.1 Study population

**Figure 1:**
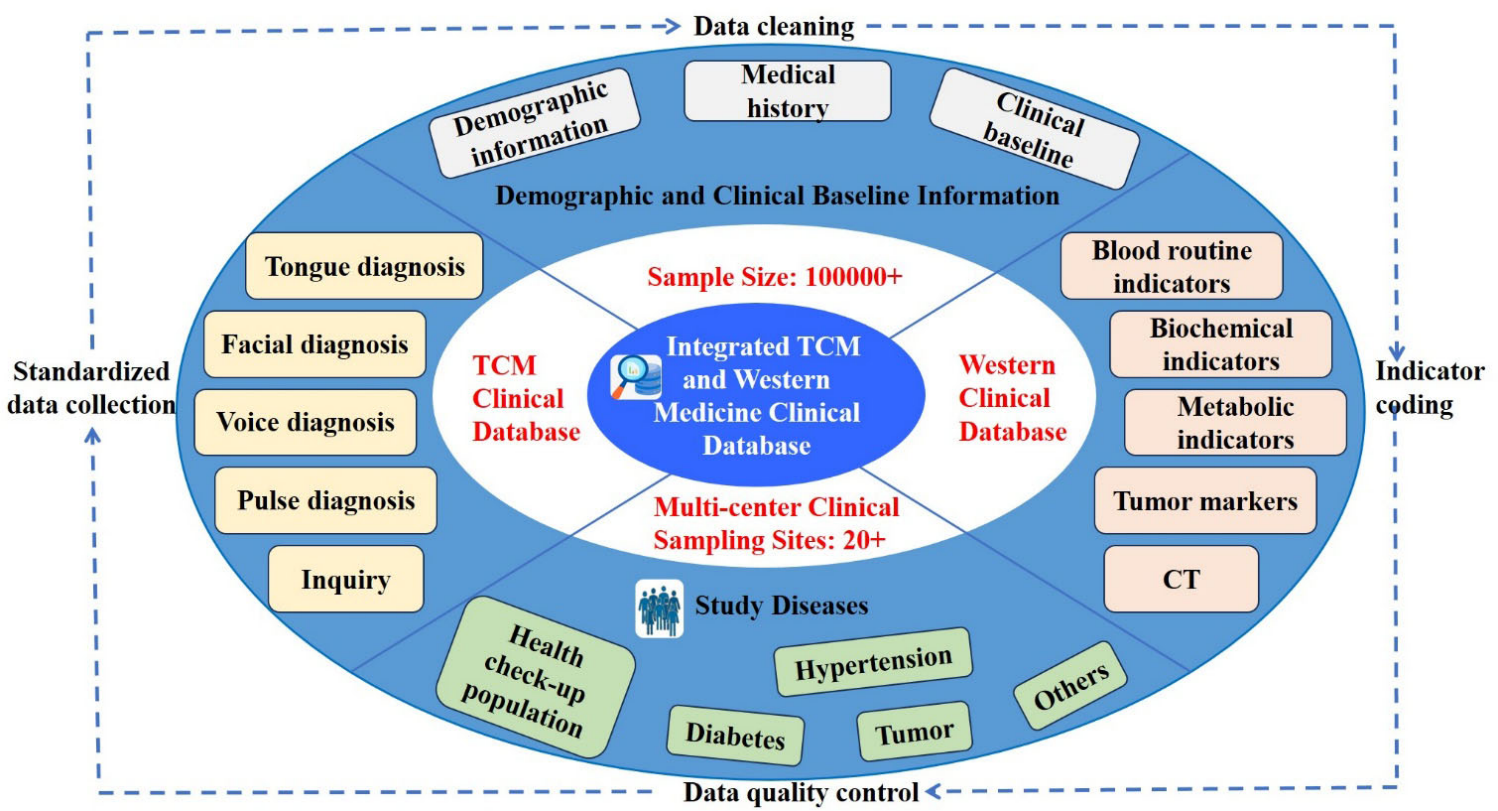
A multi-center integrated TCM and Western medicine clinical database constructed under the national key R&D program (with 100000+ samples).

The data of the present study were derived from the big data cloud platform for intelligent diagnosis constructed by the project Research and Development of an Intelligent TCM Tongue Diagnosis System (2017YFC17033301), which is a subproject of the National Key R&D Program of China during the 13th Five-Year Plan Period (**Figure 1**). Since January 2018, this platform has conducted standardized data collection at more than 30 clinical multicenter sampling sites nationwide, with a cumulative sample size of over 100,000 cases to date. The enrolled subjects cover both healthy physical examination populations and patients with various diseases, including hypertension, diabetes, lung cancer (as the primary malignant tumor), coronary heart disease, and stroke. The platform integrates dual databases of TCM and Western medicine: the TCM database contains information of the four diagnostic methods in TCM, namely inspection (including tongue diagnosis and facial diagnosis), auscultation and olfaction (including voice diagnosis), inquiry, and palpation (including pulse diagnosis); the Western medicine database includes laboratory test data such as blood routine, biochemical indicators, and tumor markers.

In this study, subjects enrolled between January 1, 2018 and December 31, 2024 were screened from the aforementioned platform, including 1315 individuals with benign pulmonary nodules (n=1315) diagnosed at the Medical Examination Center of Shuguang Hospital Affiliated to Shanghai University of Traditional Chinese Medicine (SHUTCM, Hospital A), and 1138 patients with lung cancer (n=1138) diagnosed at Longhua Hospital Affiliated to SHUTCM (Hospital B) and Yueyang Integrated Traditional Chinese and Western Medicine Hospital Affiliated to SHUTCM (Hospital C) during the same period. Their facial images were selected for subsequent analysis. All facial images of the subjects were acquired using the self-developed TFDA-1 digital tongue and facial diagnosis instrument of our research team. To further validate the generalization ability of the constructed models, an additional cohort was enrolled in this study, comprising 568 lung cancer patients recruited from Shanghai Pulmonary Hospital Affiliated to Tongji University (Hospital D) between January 1, 2025 and December 30, 2025, and 694 individuals with benign pulmonary nodules diagnosed at the Medical Examination Center of Shuguang Hospital Affiliated to SHUTCM (Hospital A) between January 1, 2021 and December 30, 2021.

### 2.2 Diagnostic criteria

#### 2.2.1 Diagnostic criteria for benign pulmonary nodules

(1) Pathological Gold Standard: Pathological confirmation of benign pulmonary lesions via surgical resection, percutaneous needle biopsy, or bronchoscopic biopsy, with no malignant tumor cells detected.(2) Imaging Diagnostic Criteria: In accordance with Guidelines for Management of Incidental Pulmonary Nodules Detected on CT Images: From the Fleischner Society (2017) (MacMahon et al., 2017), comprehensive evaluation of nodule morphology, density, dynamic changes and other indicators; tumor markers (CEA, NSE, CYFRA21-1, etc.) persistently within the normal reference range; no or extremely low high-risk factors for lung cancer; and no history of malignant tumors.

#### 2.2.2 Diagnostic criteria for the lung cancer group

Pathological Gold Standard: Pathological confirmation of primary bronchogenic carcinoma, with exclusion of pulmonary metastatic carcinoma and pulmonary invasion by tumors at other sites. The diagnostic criteria were formulated with reference to the National Comprehensive Cancer Network (NCCN) Clinical Practice Guidelines for Lung Cancer Screening (2024 Edition) (Riely et al., 2024) and the World Health Organization (WHO) Histological Classification of Lung Tumors (Nicholson et al., 2022).

### 2.3 Inclusion and exclusion criteria

#### 2.3.1 Inclusion criteria for the benign pulmonary nodule group

(1) Aged ≥18 years.(2) Solitary or multiple pulmonary nodules (maximum diameter ≤30 mm) detected by chest CT examination.(3) Meeting any of the following criteria: ① Benign nature confirmed by pathological examination; ② Typical benign imaging features (e.g., calcification, fat density, etc.); ③ Solid nodules with ≥2 years of follow-up or ground-glass nodules (GGNs) with ≥5 years of follow-up, stable with no enlargement.(4) Voluntarily participating in the study with a signed informed consent form and good clinical compliance.

#### 2.3.2 Inclusion criteria for the lung cancer group

(1) Aged ≥18 years. (2) Conforming to the aforementioned diagnostic criteria for lung cancer, with pathological confirmation of primary lung cancer and no restrictions on TNM staging. (3) No severe organ failure involving the heart, liver, kidneys or other vital organs; capable of tolerating basic clinical examinations and evaluations; with a signed informed consent form.

#### 2.3.3 Exclusion criteria

(1) History of previous or concurrent malignant tumors; (2) Facial image quality failing to meet the technical requirements for research analysis; (3) Pregnant or lactating women; (4) Diagnosed with mental disorders or with poor compliance, unable to cooperate with the study procedures; (5) For subjects with benign pulmonary nodules: Pulmonary nodules with suspected malignant features (e.g., accompanied by lobulation, spiculation, or rapid enlargement) without pathological confirmation of benignity; or complicated with severe pulmonary diseases (active pulmonary tuberculosis, pulmonary fibrosis, severe pneumonia) or autoimmune diseases; (6) For lung cancer subjects: Severe cardiopulmonary dysfunction, unable to tolerate diagnostic and study-related procedures.

## 3 Methodology

### 3.1 Clinical information collection and feature extraction

**Figure 2:**
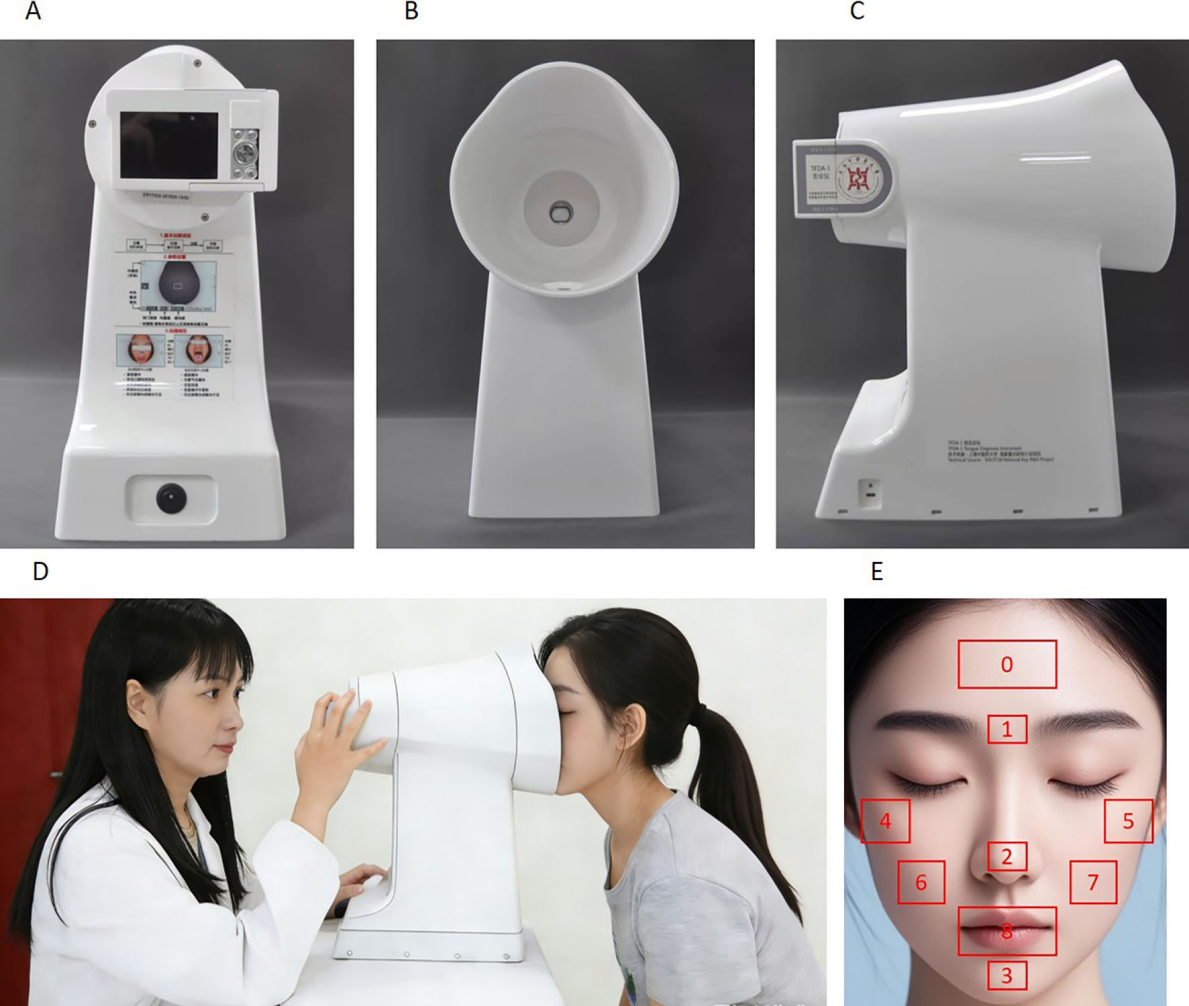
Multi-view appearance, image acquisition scenario and schematic diagram of facial image segmentation of the TFDA-1 digital tongue and facial diagnosis instrument. **(A)** Front view of the TFDA-1 digital tongue and facial diagnosis instrument; **(B)** Internal morphology of the acquisition chamber; **(C)** Lateral view of the instrument; **(D)** Practical operation scenario of image acquisition with the device; **(E)** Schematic diagram of regional segmentation and annotation of facial images.

Facial images were acquired using the TFDA−1 Digital Tongue and Face Diagnosis Instrument, which was independently developed by the research team at the Shanghai Key Laboratory of Health Identification and Assessment. This device has been certified as a Class II medical device in China (certificate number: 20212200604). To ensure high consistency and standardization across acquisitions, all instrumental parameters including shutter speed, aperture, ISO sensitivity, and light source intensity were strictly fixed. During the sample collection period, we regularly calibrated the collection equipment and standardized the color and corrected the color constancy of the tongue images to reduce systematic deviations and color drift caused by multiple devices and long-term use. In addition, the equipment was verified by standard color cards and repeated quality control, which can effectively ensure that the color measurement errors of each collection point equipment are within the allowable range. The TFDA-1 digital tongue and facial diagnosis instrument (**Figures 2A–C**), the standard facial image acquisition scenario (**Figure 2D**), and the sample facial images (**Figure 2F**) are presented in **Figure 2**.

### 3.2 Facial image acquisition protocol

(1) Device Startup and Parameter Verification Start the acquisition device and set the parameters of the tongue and facial diagnosis instrument as follows: shutter speed of 1/125 s, aperture value of F6.3, ISO sensitivity of 200, and metering mode set to center-weighted metering. (2) Device Sanitization and Disinfection Wipe the contact surface of the instrument’s acquisition port with 75% alcohol cotton balls or pads for sanitization and disinfection to prevent cross-contamination. (3) Subject Positioning and Acquisition Operation Instruct the subjects to sit upright and adjust the height of the instrument or the seat to align the face directly with the acquisition port with the head in a natural relaxed position. Guide the subjects to keep their eyes and mouth naturally closed, with facial muscles relaxed and no frowning. Observe the exposure indicator in the rectangular area on the right side of the instrument; when the indicator value reaches 0 or ±1, tap the central facial area on the touch interface, and the instrument will capture facial images automatically. Acquisition Requirements: The subject’s facial skin shall be clean, unmarked and free of makeup; no hair shall cover the forehead in the image; and facial features shall be clearly identifiable. (4) Image Quality Verification Verify the image quality in the preview window immediately after acquisition to ensure a clear image with a centered face, naturally closed eyes and mouth, an unobstructed forehead, a relaxed facial expression, and no overexposure or underexposure. If the image fails to meet the quality criteria, instruct the subject to rest for 2 minutes before re-acquisition.

### 3.3 Facial feature description

#### 3.3.1 Face detection and regional segmentation

In this study, the built-in face detection module of the third-party open-source library Dlib was adopted to achieve accurate localization and extraction of the facial region. As a core component of the Dlib library in the field of computer vision, this module is developed based on the C++ underlying layer and provides a Python calling interface, featuring high efficiency and ease of use. It can accurately output the coordinates of the rectangular facial region, which provides reliable technical support for subsequent facial feature extraction and input preprocessing of the predictive model, ensuring the input accuracy of the subsequent predictive model (Kazemi and Sullivan, 2014; King, 2009). On the basis of completing facial region localization, and in accordance with the traditional theory of facial region division in TCM facial complexion diagnosis, the human face was divided into 9 key regions in this study, namely the forehead, glabellar region, nasal tip, mandible, left zygomatic region, right zygomatic region, left cheek, right cheek, and lip region. The positions of each region refer to the sample diagram (**Figure 1F**).

#### 3.3.2 Facial image feature extraction

In this study, the extracted facial image features were mainly divided into two categories: color features and texture features. The extraction of color features covered the 9 key facial regions divided above, and the component parameters of each region in four commonly used color spaces (RGB (Kim et al., 2018), HSV (Giuliani, 2022; Kim et al., 2018), Lab (Fairchild, 2013), and YCrCb (Kim et al., 2018)) were collected respectively. The selection of multiple color spaces can comprehensively capture information such as hue, saturation, and brightness of facial skin color, avoid misjudgment of skin color features caused by a single color space, and meet the observation requirements for the depth, brightness, and luster of facial complexion in TCM facial complexion diagnosis.

The GLCM is a second-order statistical feature that provides information about texture. GLCM investigates the spatial relationships between pixels and defines the frequency of pixel combinations in specific directions and distances. For texture analysis, multiple features such as Angular Second Moment (ASM), Contrast, Entropy, and Inverse Difference Moment (IDM) can be calculated through GLCM (Manav et al., 2023; Sahinis and Kellis, 2023). Previous studies have validated that GLCM texture features can effectively extract pathological information from biomedical signals. Combined with CNNs and SVM, they enable automatic diagnosis of myocardial infarction, demonstrating the feasibility and effectiveness of GLCM in non-invasive biomedical signal diagnosis (Attallah and Ragab, 2023). To comprehensively capture facial texture information, texture feature extraction adopted a combination of multi-angle and fixed distance in this study: four directions (0°, 45°, 90°, 135°) and a fixed distance were selected, ultimately generating a total of 16 texture feature indicators, namely Asm_0, Asm_1, Asm_2, Asm_3, Con_0, Con_1, Con_2, Con_3, Idm_0, Idm_1, Idm_2, Idm_3, Ent_0, Ent_1, Ent_2, and Ent_3.

### 3.4 Feature selection and model development and validation process

#### 3.4.1 Feature selection

This study employed a multi-step feature selection strategy to construct a robust core feature set while reducing feature redundancy, instability, and overfitting risks. Firstly, constant features with a variance of zero were eliminated using the variance threshold method; then, features with a Pearson correlation coefficient (|r|) greater than 0.95 were removed to avoid unstable parameter estimation in subsequent modeling. Subsequently, a logistic regression model was constructed using lung cancer and benign pulmonary nodules as outcome variables, and slight Gaussian perturbations were added to the training set to evaluate feature robustness. The odds ratio (OR), 95% confidence interval (CI), and P value of each feature were calculated one by one. To retain statistically robust features with potential clinical interpretability, the following selection criteria were set: (1) Eliminate features with extremely low OR values (<0.001 or >1000), as these features have extremely unstable effect estimates under minor data perturbations and poor clinical interpretability; (2) Eliminate features with overly wide confidence intervals (the ratio of the upper and lower bounds of the CI is >100), as these features have insufficient statistical accuracy and unreliable results; (3) Eliminate features with a confidence interval crossing 1, no statistical significance, or having numerical anomalies. Features that met all the selection criteria were included in the final core feature set. To avoid overfitting when performing feature selection on the same dataset, the entire selection process was completed within a 10-fold cross-validation framework.

#### 3.4.2 Model development and validation process

The original dataset was divided into a training set (80%) and an internal test set (20%) at an 8:2 ratio using stratified sampling to ensure consistent class distribution across subsets. An independent external validation set was also introduced to comprehensively verify the generalization ability of the models. The detailed process of model development and validation is as follows:

#### 3.4.3 Data preprocessing

Outlier handling: For continuous variables following a normal distribution, outliers were identified using the Z-Score method (|Z|>3) and replaced with the mean value. For non-normally distributed variables, the Interquartile Range (IQR) method was adopted for outlier identification, with Q1−1.5×IQR and Q3 + 1.5×IQR as the lower and upper bounds; outliers beyond this range were replaced with the median value.

Missing value handling: Appropriate imputation methods were selected based on the distribution type of variables: the mean value was used for normally distributed variables, and the median value for non-normally distributed variables.

#### 3.4.4 Model training and cross-validation

Four core machine learning models were trained on the training set, including eXtreme Gradient Boosting (XGBoost), Light Gradient Boosting Machine (LightGBM), Support Vector Machine (SVM), and Gradient Boosting Decision Tree (GBDT). A stratified 10-fold cross-validation framework was employed to evaluate model stability, with the specific process as follows: the training set was equally divided into 10 subsets; iterative training and validation were performed 10 times, with 9 subsets serving as the sub-training set and 1 subset as the validation set in each round, ensuring all data participated in model validation. In each round of validation, the model was trained to output predicted probabilities, and the Receiver Operating Characteristic (ROC) curve, Precision-Recall (PR) curve, calibration curve, and decision curve were calculated and plotted. The results of the 10 validation rounds were aggregated, and the average curve of each metric was obtained via point-by-point averaging, with ±1 standard deviation (SD) used to characterize the curve fluctuation. This provided quantitative and visual evidence for the comparison of model performance.

#### 3.4.5 Hyperparameter optimization

Taking the average AUC obtained from stratified 10-fold cross-validation as the primary evaluation metric, the Grid Search algorithm was combined to perform hyperparameter optimization for the four models, and the optimal hyperparameter combination for each model was determined. The models were retrained on the complete training set with the optimal hyperparameters to fully explore the inherent patterns of the data and fix the model parameters. Meanwhile, the details of the optimal hyperparameters for each model were saved to ensure the reproducibility of the modeling process.

#### 3.4.6 Internal test set evaluation

The four models with fixed parameters were applied to the internal test set that was not involved in training or hyperparameter tuning for a comprehensive performance evaluation. The evaluation metrics included Accuracy, Precision, Sensitivity, Specificity, F1-Score, AUC, Average Precision (AP), and Brier Score. Visualization charts for individual and multi-model comparison (ROC curve, PR curve, calibration curve, decision curve, and confusion matrix) were also plotted to systematically characterize the discriminative performance and clinical utility of each model.

The calibration curve (Ziegler, 2020) was used to evaluate the consistency between the model-predicted probabilities and the actual occurrence probabilities of events. The curve was plotted with predicted probability as the x-axis and actual occurrence rate as the y-axis, and compared with the 45° ideal calibration line. The Hosmer-Leme show test and Brier Score (a smaller value indicates a smaller prediction error) were adopted to quantify the calibration effect. The closer the curve is to the 45° ideal line, the higher the consistency between the model-predicted and actual probabilities, and the better the calibration performance.

Decision Curve Analysis (DCA) (Vickers and Elkin, 2006; Vickers et al., 2019) is an important method for evaluating the clinical utility of predictive models. By calculating the net benefit of the model at different decision thresholds and comparing it with two extreme strategies (Treat All and Treat None), the actual value of the model in assisting clinical decision-making was quantified, so as to judge the potential of the model for clinical promotion and application.

### 3.5 External validation

In accordance with the Model Locking principle, the four models were directly applied to a completely independent external validation set (with data from different periods and sampling sites). The same evaluation metrics and visualization methods as those for the internal test set were adopted. In addition, a comparative analysis of modeling features between the development cohort and the external validation set was newly added to systematically evaluate the discriminative performance, calibration accuracy, generalization ability, and clinical application value of each model in the novel dataset. The comparison results of model performance between the internal and external test sets were generated accordingly.

### 3.6 Model interpretability analysis

In this study, SHapley Additive exPlanations (SHAP) method was used for model interpretability analysis. The rank plot of mean absolute SHAP values was used to evaluate the contribution of each feature to the model prediction results. Meanwhile, beeswarm plots, decision plots, and heat maps were comprehensively applied for the visualization of feature effects, systematically presenting feature importance, feature value distribution, the positive and negative effects of features on predictions, and the distribution characteristics of SHAP values (Deng et al., 2022; Lundberg and Lee, 2017). Waterfall plots (Rahmani et al., 2024) were used to decompose the prediction process of individual samples, clearly showing the contribution and influence direction of each feature on the prediction result of the sample. In addition, dependence plots and interaction scatter plots were used to further explore the nonlinear interaction relationships among the top three key features and their modulatory effects on the model prediction results (Deng et al., 2022).

**Figure 3:**
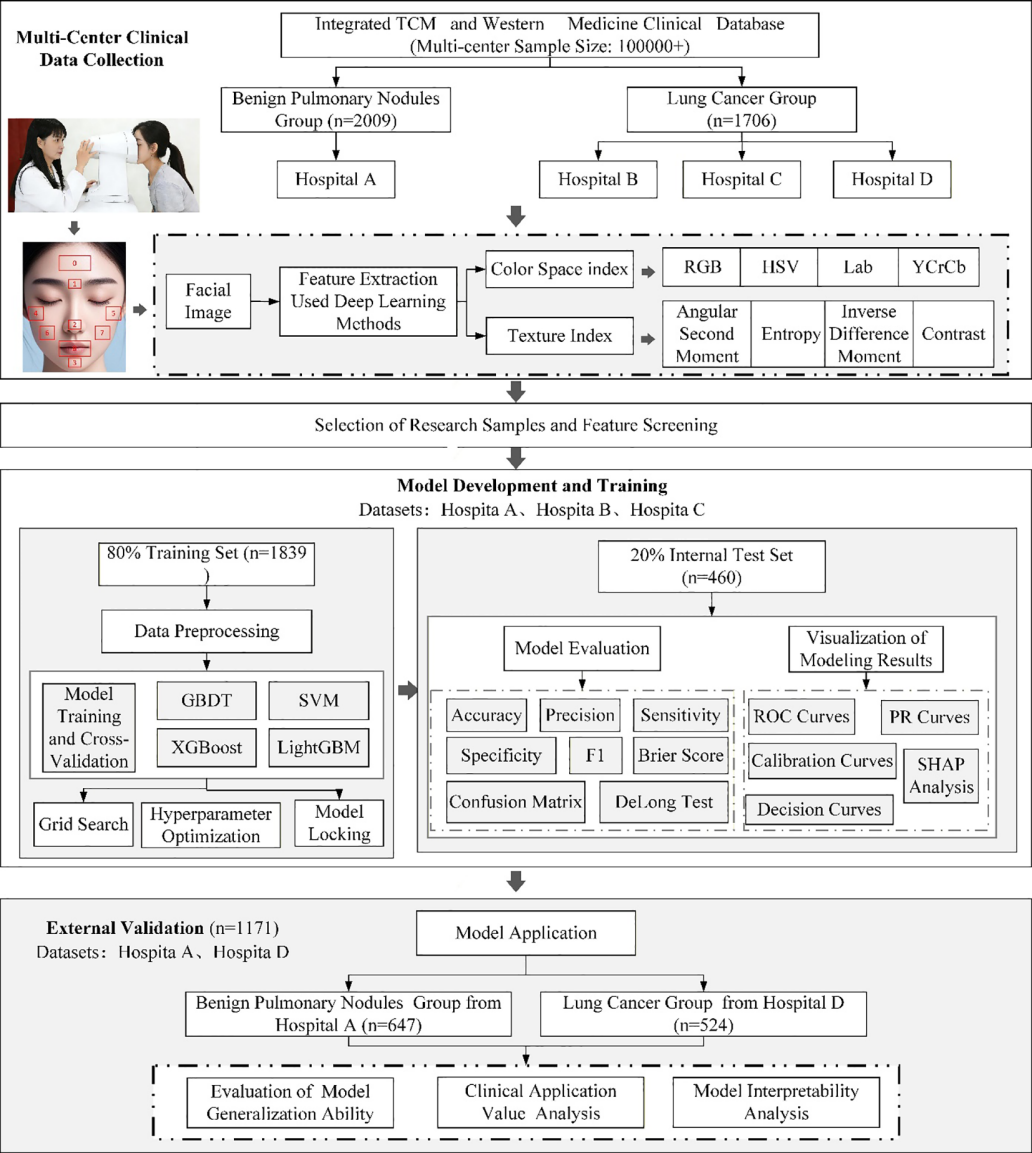
Schematic workflow of the multi-center study on facial-image-based lung cancer prediction.

The technical roadmap of this study is presented in **Figure 3**.

### 3.7 Statistical analysis

All statistical analyzes of the data were performed using Python software. Categorical data were statistically described by frequency (constituent ratio, %), and the Chi-square (χ²) test was used for intergroup comparisons; if the applicable conditions of the test were not met, Fisher’s exact test was performed instead. For continuous data, normality tests were first conducted: data conforming to a normal distribution were expressed as mean ± standard deviation (x̄ ± SD), while non-normally distributed data were expressed as median (interquartile range) [M (Q1, Q3)]. For intergroup comparisons of continuous data, the independent samples t-test was adopted if the assumptions of normality and homogeneity of variance were satisfied; otherwise, the Mann-Whitney U nonparametric rank-sum test was used. In the correlation analysis between indicators, Pearson correlation analysis was performed for indicators conforming to a bivariate normal distribution, and Spearman’s rank correlation analysis was used for non-normally distributed indicators. All statistical tests were two-tailed, with a significance level of α=0.05; a *P*-value < 0.05 was considered statistically significant.

## 4 Experiments and results

### 4.1 Baseline characteristics of study participants

After screening in accordance with the inclusion and exclusion criteria, a total of 2299 participants were enrolled in the model training and development phase, including 1275 cases in the benign pulmonary nodule group and 1024 cases in the lung cancer group. A total of 1171 participants were included in the model external validation phase, comprising 647 cases in the benign pulmonary nodule group and 524 cases in the lung cancer group. Comparisons of the baseline characteristics of participants across groups are detailed in **Tables 1**, **2**.

**Table 1:** Baseline characteristics and intergroup comparisons of participants in the training and development dataset.

Characteristic		Training and development dataset		Statistical test	
		Benign pulmonary nodule group	Lung cancer group	χ²/Z	*P* value
Gender (n, %)	Male	550 (43.14)	566 (55.27)	44.68	< 0.001
	Female	725 (56.86)	458 (44.73)		
Age [M(Q1,Q3)]		46 (37,56)	66 (56,71)	-28.038	< 0.001

**Table 2:** Baseline characteristics and intergroup comparisons of participants in the external validation set.

Characteristic		External validation set		Statistical test	
		Benign pulmonary nodule group	Lung cancer group	χ²/Z	*P* value
Gender (n, %)	Male	334(51.62)	373(71.18)	67.649	< 0.001
	Female	313(48.38)	151(28.82)		
Age [M(Q1,Q3)]		42(34,51)	66(59,71)	-28.9114	< 0.001

Statistical analysis results showed that in both the training and development dataset and the external validation dataset, the gender and age distributions between the benign pulmonary nodule group and the lung cancer group exhibited statistically significant differences (all *P* < 0.001). Both datasets showed consistent characteristics: the proportion of males in the lung cancer group was significantly higher than that in the benign pulmonary nodule group, and the median age of the lung cancer group was significantly higher.

### 4.2 Feature selection based on logistic regression

**Figure 4:**
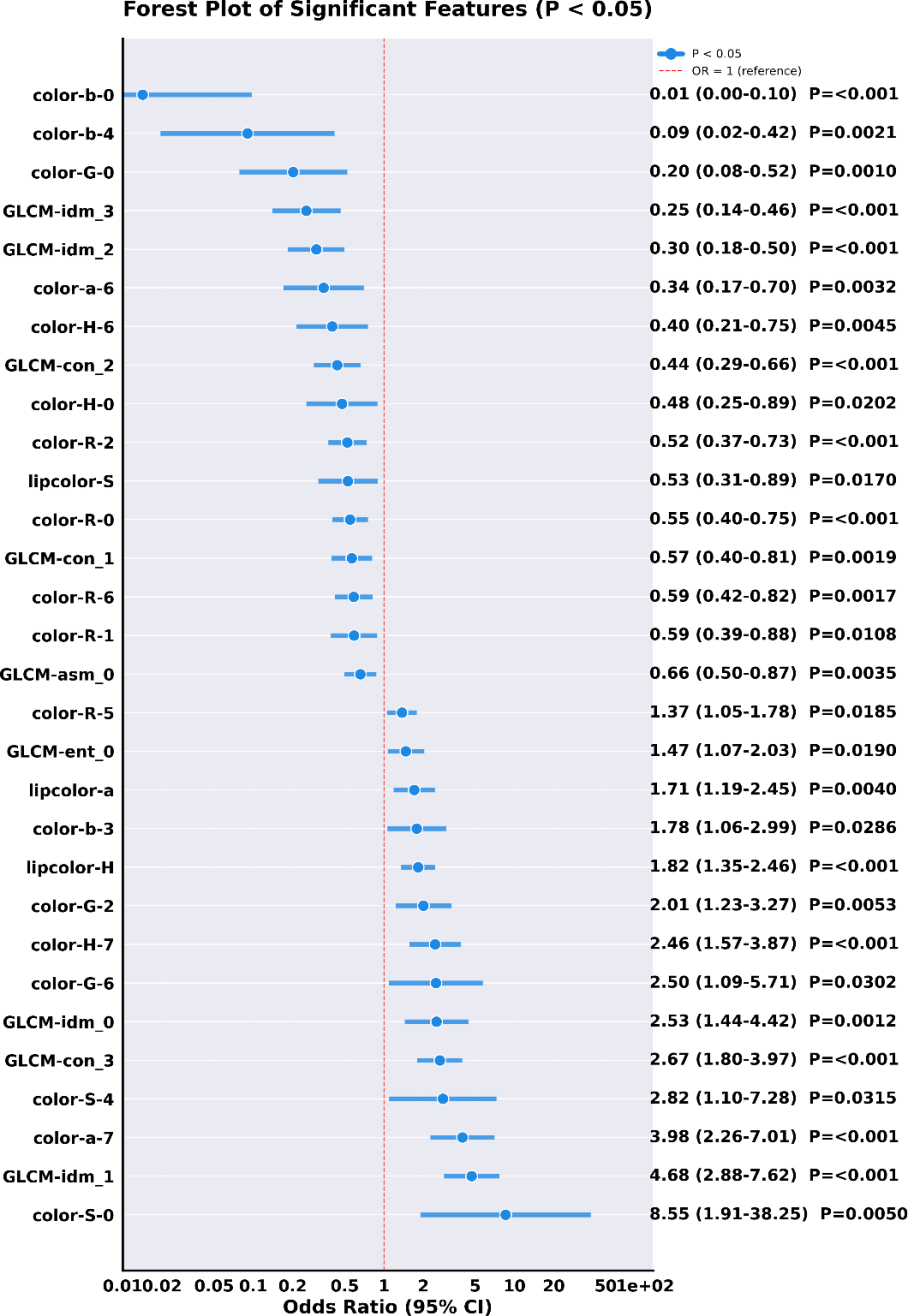
Forest plot of statistically significant facial features.

Based on logistic regression analysis, this study screened out 70 valid features from 124 facial image features; the forest plot of their odds ratio (OR) values and 95% confidence intervals (CIs) was shown in **Supplementary Figure 1**. Among these characteristics, 30 of them were statistically significant (P<0.05). The OR values and 95% confidence intervals were presented in **Table 3**, and the corresponding forest plot was shown in **Figure 4**. These 30 features were included in subsequent correlation analysis and the construction of machine learning early warning models for lung cancer.

**Table 3:** OR and 95% CI of each feature index based on logistic regression analysis.

Feature	Coefficient	Standard error	OR	95% CI	*P* value
lipcolor-H	0.6011	0.152	1.824	[1.3540, 2.4572]	0.0001
lipcolor-S	-0.6358	0.2665	0.5295	[0.3141, 0.8927]	0.017
lipcolor-a	0.5339	0.1857	1.7056	[1.1854, 2.4542]	0.004
GLCM-asm_0	-0.4186	0.1433	0.658	[0.4968, 0.8714]	0.0035
GLCM-ent_0	0.3865	0.1648	1.4718	[1.0655, 2.0331]	0.019
GLCM-idm_0	0.9262	0.2858	2.525	[1.4421, 4.4213]	0.0012
GLCM-con_1	-0.5689	0.1829	0.5661	[0.3956, 0.8102]	0.0019
GLCM-idm_1	1.5433	0.2484	4.6799	[2.8759, 7.6156]	0
GLCM-con_2	-0.8247	0.2115	0.4384	[0.2896, 0.6636]	0.0001
GLCM-idm_2	-1.1943	0.2551	0.3029	[0.1837, 0.4993]	0
GLCM-con_3	0.9838	0.2016	2.6746	[1.8016, 3.9706]	0
GLCM-idm_3	-1.369	0.3072	0.2544	[0.1393, 0.4644]	0
color-R-0	-0.5971	0.161	0.5504	[0.4015, 0.7546]	0.0002
color-G-0	-1.5985	0.4862	0.2022	[0.0780, 0.5243]	0.001
color-H-0	-0.7421	0.3195	0.4761	[0.2545, 0.8905]	0.0202
color-S-0	2.1459	0.7644	8.5501	[1.9113, 38.2484]	0.005
color-b-0	-4.2561	0.9845	0.0142	[0.0021, 0.0976]	0
color-R-1	-0.5293	0.2076	0.589	[0.3922, 0.8848]	0.0108
color-R-2	-0.6456	0.1719	0.5244	[0.3744, 0.7344]	0.0002
color-G-2	0.6966	0.2496	2.0069	[1.2305, 3.2730]	0.0053
color-b-3	0.5782	0.2642	1.7829	[1.0623, 2.9924]	0.0286
color-S-4	1.0384	0.4828	2.8247	[1.0965, 7.2765]	0.0315
color-b-4	-2.4067	0.7823	0.0901	[0.0194, 0.4175]	0.0021
color-R-5	0.3147	0.1336	1.3698	[1.0542, 1.7800]	0.0185
color-R-6	-0.5351	0.1708	0.5856	[0.4190, 0.8184]	0.0017
color-G-6	0.9144	0.4219	2.4953	[1.0914, 5.7054]	0.0302
color-H-6	-0.9115	0.3208	0.4019	[0.2143, 0.7538]	0.0045
color-a-6	-1.0653	0.3616	0.3446	[0.1697, 0.7000]	0.0032
color-H-7	0.901	0.2304	2.462	[1.5672, 3.8675]	0.0001
color-a-7	1.3819	0.2881	3.9826	[2.2642, 7.0050]	0

### 4.3 Correlation analysis results of facial image features between the two groups

**Figure 5:**
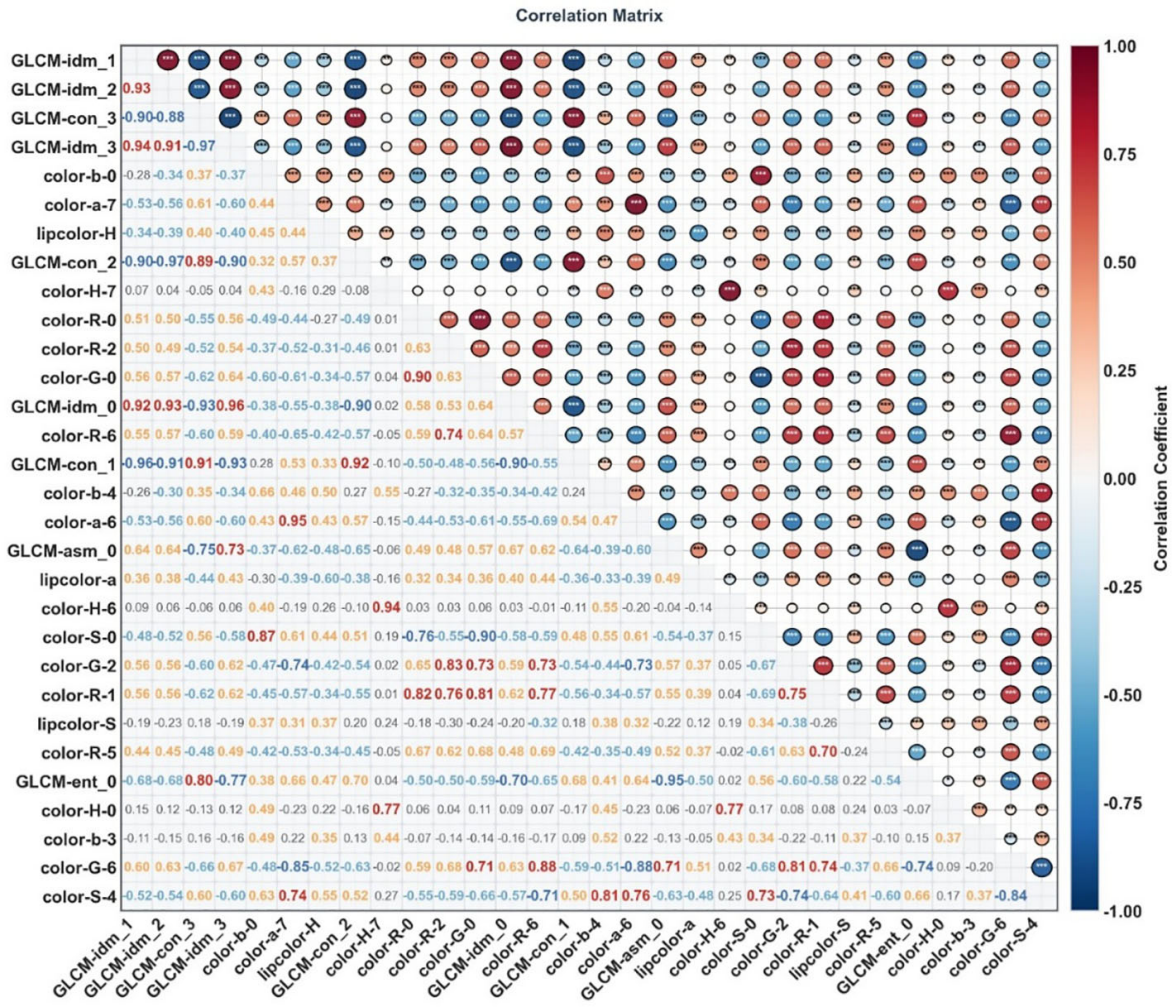
Correlation heat map of facial imaging features in patients with benign pulmonary nodules. *P < 0.05, **P < 0.01, ***P < 0.001.

**Figure 6:**
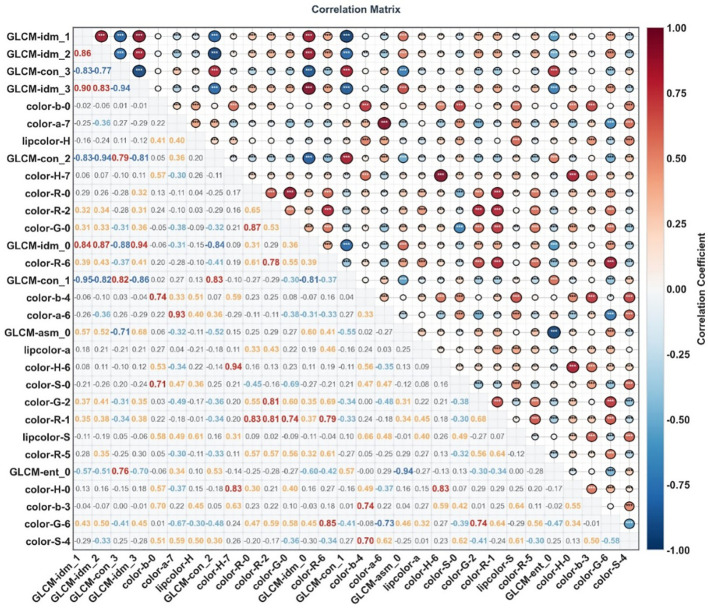
Correlation heat map of facial imaging features in patients with lung cancer. *P < 0.05, **P < 0.01, ***P < 0.001.

Correlation analysis of facial image features between the benign pulmonary nodule group and the lung cancer group was performed using Python software, and feature correlation heat maps were plotted. The correlation heat maps of facial image features for the benign pulmonary nodule group and the lung cancer group are presented in **Figures 5**, **6**, respectively. In the upper right triangular area of the heat map, the size of circular areas was used to characterize the correlation strength between features, and the depth and color system of colors were used to reflect the correlation direction. Meanwhile, asterisks indicating corresponding statistical significance were marked based on *P*-values. In the lower left triangular area of the heat map, the specific correlation coefficient values between features were labeled within rounded rectangular boxes.

The study found that the core combinations of facial image features in both the benign pulmonary nodule group and the lung cancer group exhibited significant correlations: Among GLCM texture features, an extremely strong negative correlation was observed between GLCM-idm and GLCM-con series features; among color features, intra-channel features showed an extremely strong positive correlation. Moreover, the correlation patterns of such core feature combinations were highly consistent between the two groups — among the top 5 feature combinations with extremely strong correlations in both groups, 3 were completely overlapping, namely GLCM-idm_1 and GLCM-con_1, GLCM-idm_2 and GLCM-con_2, and GLCM-idm_3 and GLCM-idm_0. The absolute values of the correlation coefficients of these 3 feature combinations in the benign pulmonary nodule group were all higher than those in the lung cancer group. Further analysis revealed that the absolute values of correlation coefficients of core feature combinations in the benign pulmonary nodule group were overall higher than those in the lung cancer group, while the lung cancer group had a higher proportion of feature combinations with weak correlations and very weak/no correlations.

### 4.4 Modeling results based on facial features

The distributions of modeling features in the training set and test set of the model development cohort are presented in **Table 4** below.

**Table 4:** Distributions of modeling features during the model training and development cohort.

Feature	Training set (n=1839)	Test set (n=460)	*P* value
GLCM-idm_1	0.79 (0.77-0.81)	0.79 (0.76-0.81)	0.1872
GLCM-idm_2	0.77 (0.75-0.79)	0.77 (0.74-0.79)	0.309
GLCM-con_3	0.49 (0.41-0.60)	0.51 (0.40-0.60)	0.5585
GLCM-idm_3	0.76 (0.73-0.79)	0.76 (0.73-0.79)	0.3424
color-b-0	19.72 (17.01-22.08)	19.60 (16.94-21.70)	0.3102
color-a-7	21.97 (19.60-24.25)	21.95 (19.74-24.43)	0.6855
lipcolor-H	2.36 (1.61-3.39)	2.37 (1.65-3.40)	0.8025
GLCM-con_2	0.37 (0.32-0.43)	0.37 (0.32-0.44)	0.4365
color-H-7	6.88 (5.84-7.90)	6.84 (5.77-7.85)	0.5939
color-R-0	163.87 (154.28-172.22)	163.67 (154.23-171.95)	0.6751
color-R-2	184.85 (179.74-189.64)	184.85 (179.83-189.80)	0.8546
color-G-0	105.73 (93.03-118.99)	105.71 (93.03-117.74)	0.6887
GLCM-idm_0	0.79 (0.76-0.81)	0.78 (0.76-0.81)	0.4241
color-R-6	175.31 (168.67-181.90)	174.51 (167.24-181.13)	0.0896
GLCM-con_1	0.35 (0.30-0.41)	0.36 (0.30-0.42)	0.2353
color-b-4	21.03 (18.75-23.53)	20.87 (18.64-23.09)	0.1996
color-a-6	22.22 (19.83-24.52)	22.01 (19.88-24.60)	0.9592
GLCM-asm_0	0.17 (0.14-0.21)	0.17 (0.13-0.21)	0.3122
lipcolor-a	28.09 ± 2.42	28.01 ± 2.39	0.5199
color-H-6	6.93 (5.93-7.97)	6.89 (5.93-7.93)	0.8039
color-S-0	118.53 (103.20-130.61)	118.87 (103.31-129.68)	0.7804
color-G-2	130.10 (122.22-137.13)	128.62 (121.45-137.50)	0.2631
color-R-1	175.25 (168.20-181.81)	175.39 (169.15-182.18)	0.8302
lipcolor-S	123.12 (117.49-128.79)	122.65 (117.39-127.71)	0.2502
color-R-5	163.52 (154.63-171.57)	163.52 (153.73-171.47)	0.5908
GLCM-ent_0	1.98 ± 0.33	1.99 ± 0.33	0.4221
color-H-0	7.26 (6.19-8.20)	7.17 (6.10-8.09)	0.3407
color-b-3	19.03 (16.93-21.13)	18.96 (17.01-20.75)	0.397
color-G-6	116.84 (105.07-126.90)	115.44 (104.26-126.30)	0.1918
color-S-4	124.04 (110.68-134.26)	123.63 (111.51-133.23)	0.9628

**Figure 7:**
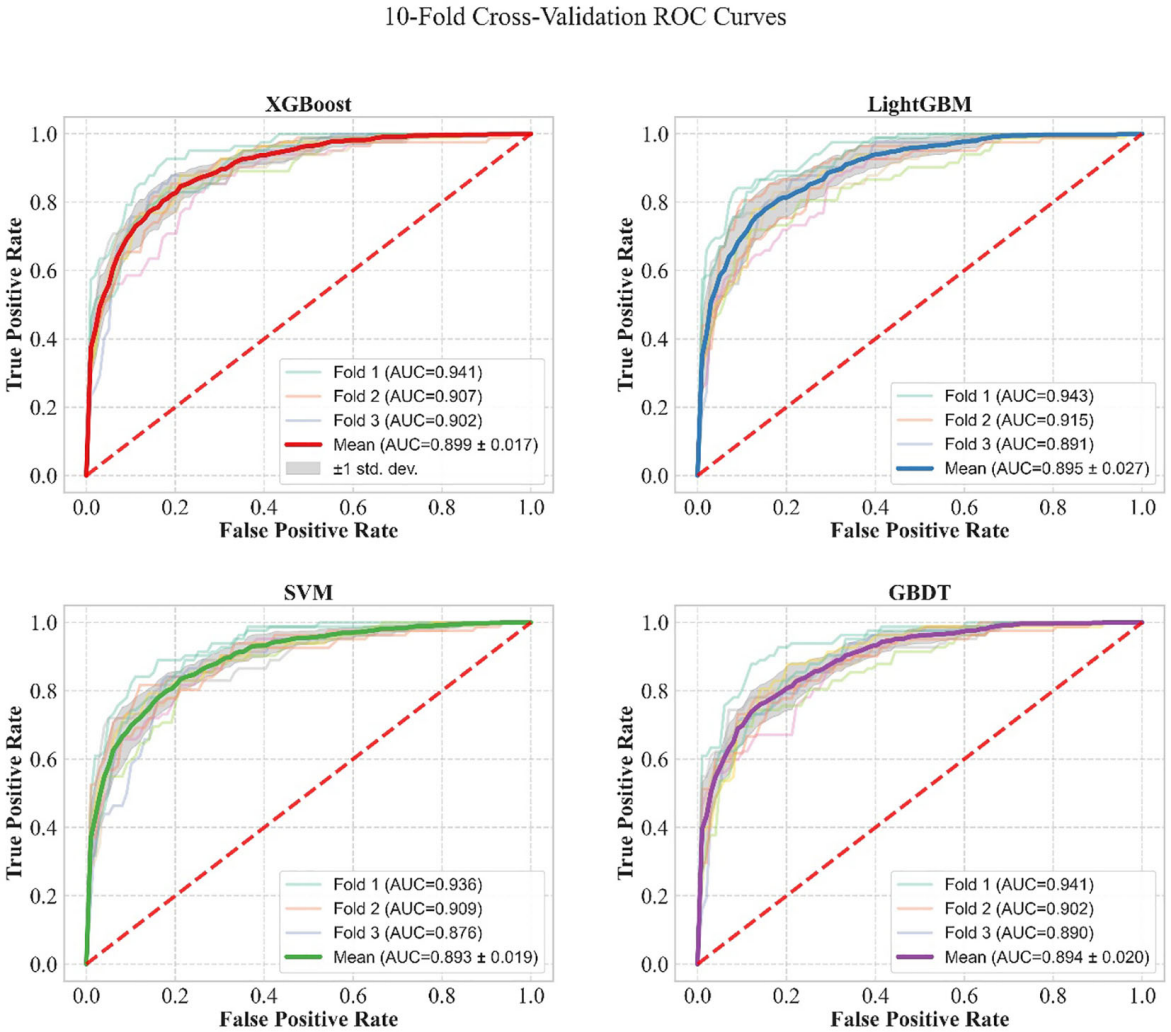
Comparison of ROC curves of the four machine learning models under 10−fold cross−validation in the training set.

**Figure 8:**
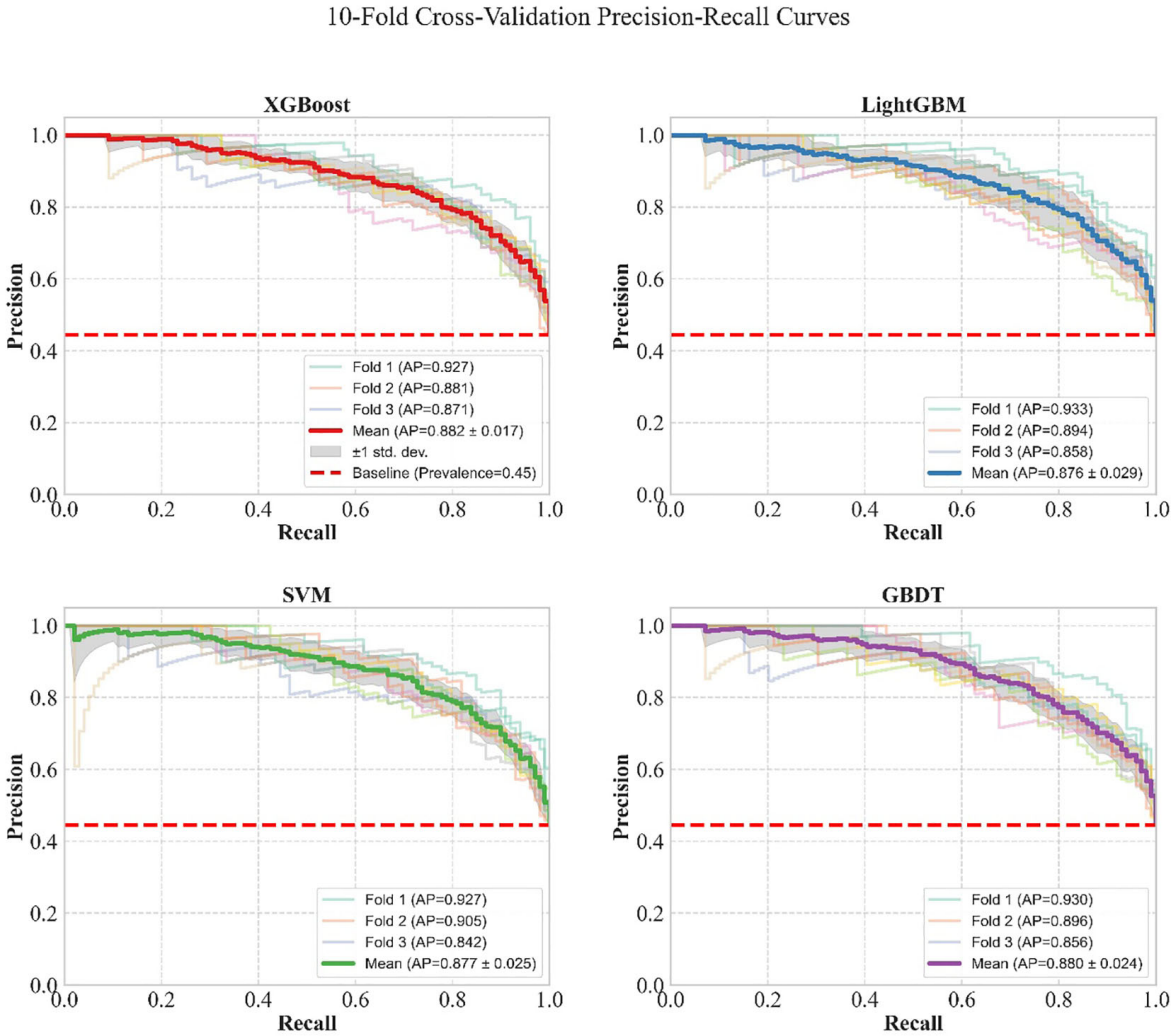
Comparison of PR curves of the four machine learning models under 10-fold cross-validation in the training set.

**Figure 9:**
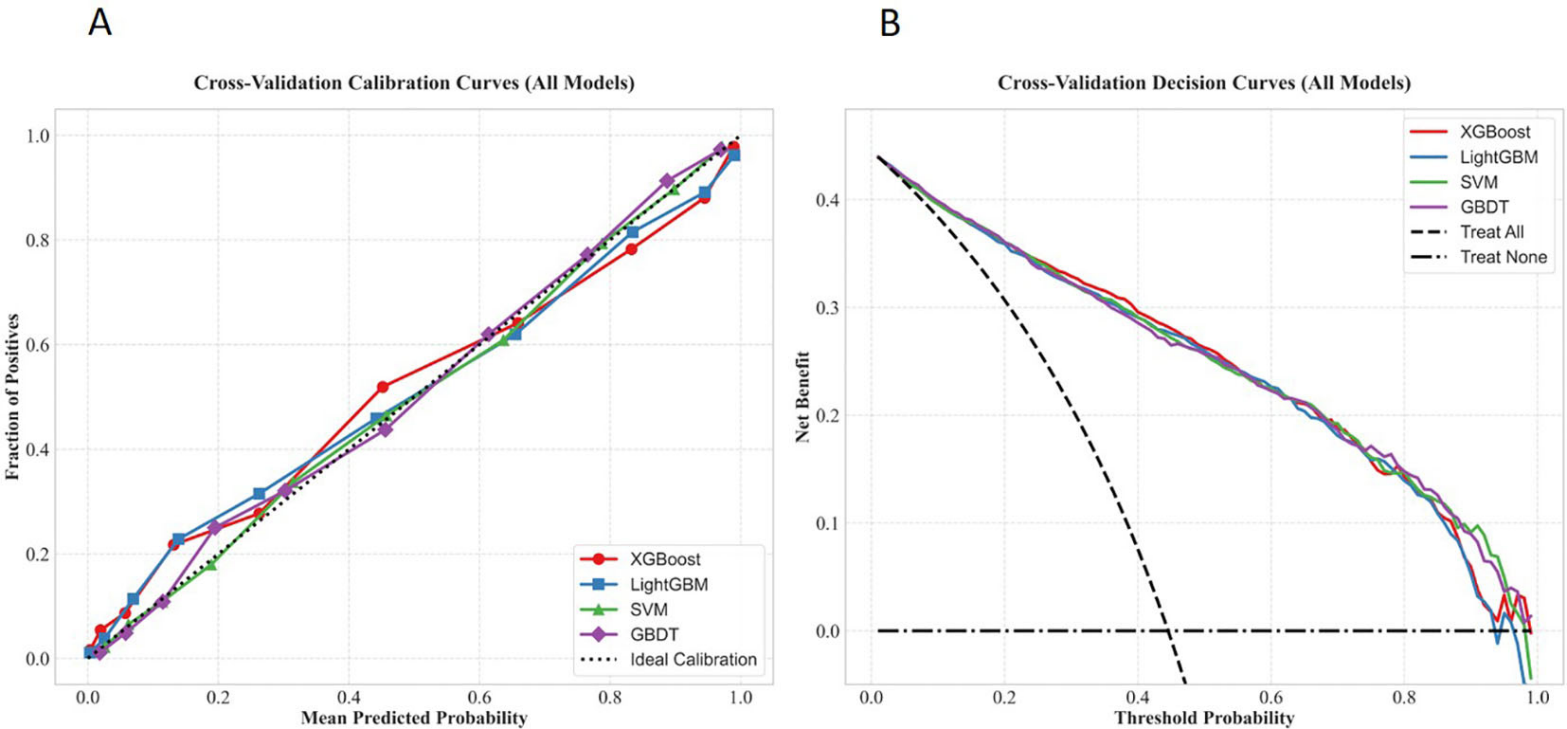
Calibration curves and decision curves of the four machine learning models under cross−validation. **(A)** Calibration curves; **(B)** Decision curves.

The results of 10-fold cross-validation for each model based on the training set, as well as the ROC curves, PR curves, calibration curves, and decision curves of each model, are detailed in **Table 5**; **Figures 7**–**9**. The model performance metrics for each fold during 10-fold cross-validation are provided in **Supplementary Table 1**.

**Table 5:** Results of 10-fold cross-validation for models based on the training set.

Model	AUC	AP	Accuracy	Precision	Sensitivity	Specificity	F1
XGBoost	0.899	0.882	0.817	0.807	0.777	0.850	0.791
LightGBM	0.895	0.876	0.814	0.811	0.761	0.857	0.785
SVM	0.893	0.877	0.807	0.782	0.787	0.824	0.784
GBDT	0.894	0.880	0.811	0.807	0.762	0.851	0.782

**Figure 10:**
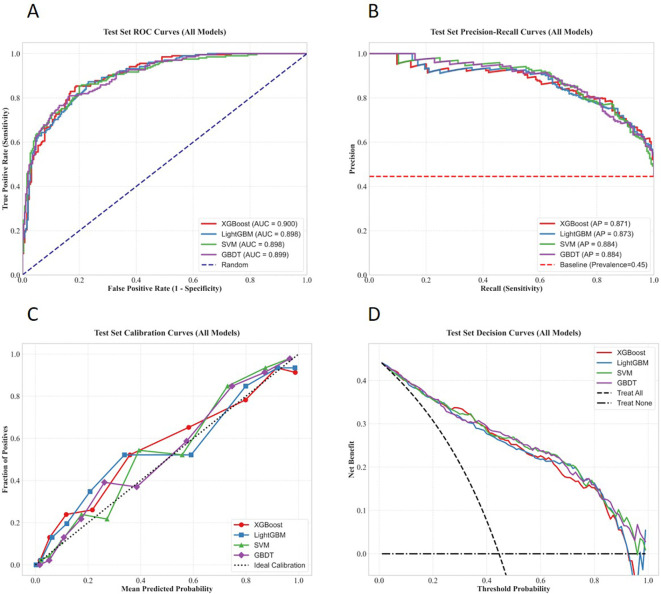
Performance evaluation curves of the four machine learning models on the internal test set. **(A)** ROC curve; **(B)** PR curve; **(C)** Calibration curve; **(D)** Decision curve.

**Figure 11:**
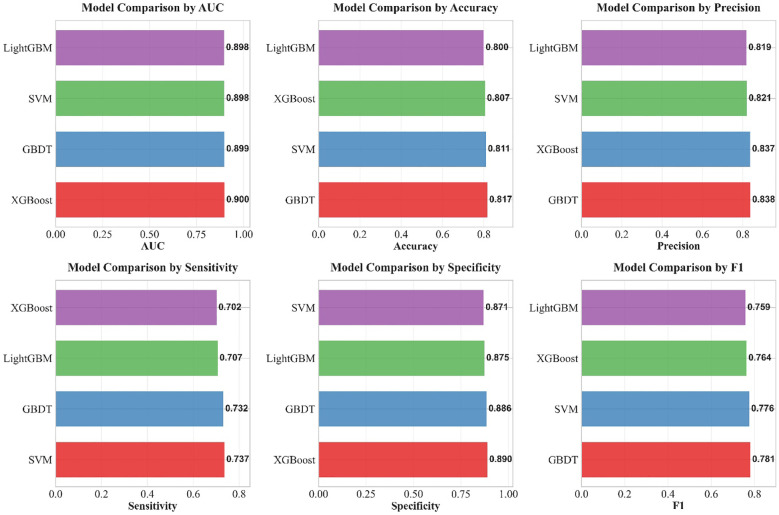
Comparison of multiple performance metrics of the four machine learning models on the internal test set.

The performance evaluation curves of each model based on the internal test set are presented in **Figures 10**, **11** below.

The distributions of modeling features in the model development cohort and external validation cohort are presented in **Table 6** below.

**Table 6:** Comparison of modeling features between the development cohort and external validation cohort.

Feature	Development cohort (n=2299)	External validation cohort (n=1171)	*P* Value
GLCM-idm_1	0.79 (0.77-0.81)	0.78 (0.76-0.81)	0.0014
GLCM-idm_2	0.77 (0.75-0.79)	0.76 (0.73-0.78)	0.0000
GLCM-con_3	0.50 (0.41-0.60)	0.50 (0.42-0.62)	0.0262
GLCM-idm_3	0.76 (0.73-0.79)	0.76 (0.73-0.79)	0.0140
color-b-0	19.68 (17.00-22.03)	20.33 (17.30-22.60)	0.0001
color-a-7	21.97 (19.65-24.28)	21.05 (19.04-23.18)	0.0000
lipcolor-H	2.37 (1.61-3.39)	2.71 (1.77-4.03)	0.0000
GLCM-con_2	0.37 (0.32-0.44)	0.40 (0.35-0.47)	0.0000
color-H-7	6.87 (5.83-7.89)	7.49 (6.07-8.92)	0.0000
color-R-0	163.84 (154.28-172.19)	163.29 (154.45-170.89)	0.3542
color-R-2	184.85 (179.75-189.67)	184.43 (180.60-189.27)	0.7024
color-G-0	105.73 (93.03-118.77)	107.72 (95.49-120.23)	0.0039
GLCM-idm_0	0.79 (0.76-0.81)	0.78 (0.76-0.80)	0.0223
color-R-6	175.31 (168.48-181.74)	174.97 (168.89-180.54)	0.2558
GLCM-con_1	0.35 (0.30-0.41)	0.36 (0.31-0.43)	0.0005
color-b-4	20.99 (18.74-23.43)	21.62 (19.15-24.72)	0.0000
color-a-6	22.19 (19.84-24.54)	21.50 (19.20-23.48)	0.0000
GLCM-asm_0	0.17 (0.13-0.21)	0.17 (0.13-0.20)	0.0063
lipcolor-a	28.11 (26.47-29.72)	28.10 (25.97-29.69)	0.0749
color-H-6	6.92 (5.93-7.96)	7.48 (6.21-8.91)	0.0000
color-S-0	118.56 (103.23-130.39)	117.85 (103.14-129.50)	0.6425
color-G-2	129.89 (122.00-137.23)	131.58 (124.89-138.37)	0.0000
color-R-1	175.26 (168.41-181.94)	174.70 (169.30-181.22)	0.9921
lipcolor-S	123.07 (117.48-128.58)	124.03 (119.12-129.38)	0.0003
color-R-5	163.52 (154.41-171.57)	163.06 (153.64-170.64)	0.0725
GLCM-ent_0	1.98 ± 0.33	2.00 ± 0.33	0.0826
color-H-0	7.24 (6.16-8.19)	7.94 (6.71-9.18)	0.0000
color-b-3	19.03 (16.95-21.02)	19.83 (17.24-22.34)	0.0000
color-G-6	116.60 (104.97-126.72)	117.25 (107.50-126.81)	0.0199
color-S-4	123.94 (110.89-134.09)	124.19 (111.96-134.23)	0.6619

**Figure 12:**
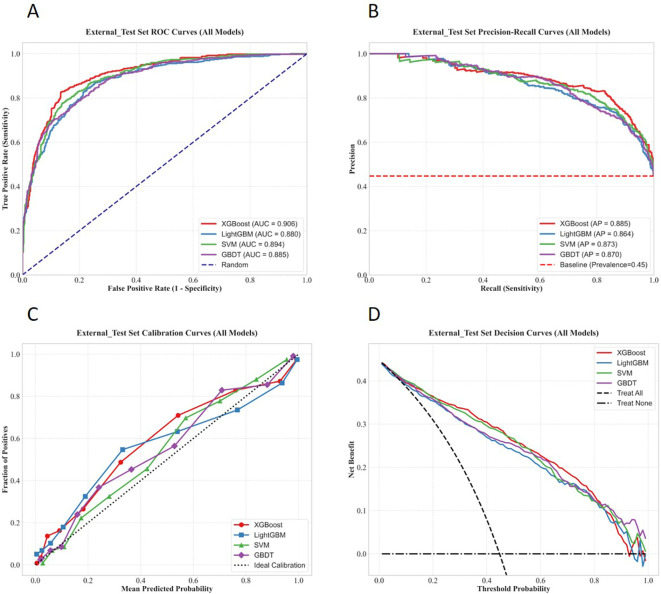
Multidimensional performance evaluation curves of the four machine learning models on the external test set. **(A)** ROC curve; **(B)** PR curve; **(C)** Calibration curve; **(D)** Decision curve.

**Figure 13:**
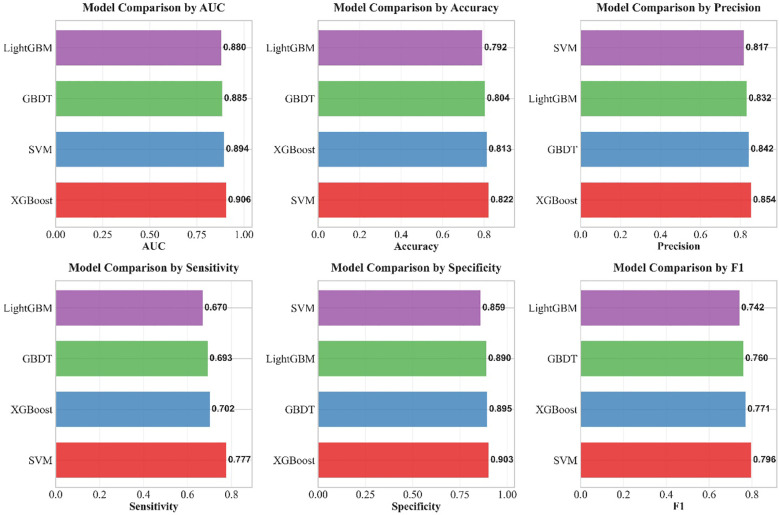
Comparison of multiple performance metrics of the four machine learning models on the external test set.

The performance evaluation curves of each model during the external validation phase are presented in **Figures 12**, **13** below.

**Figure 14:**
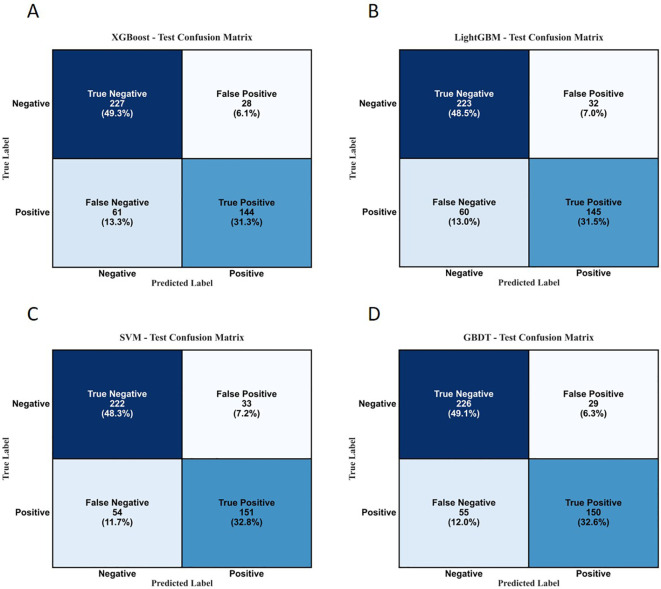
Confusion matrices of each model on the internal test set. **(A)** XGBoost; **(B)** LightGBM; **(C)** SVM; **(D)** GBDT.

**Figure 15:**
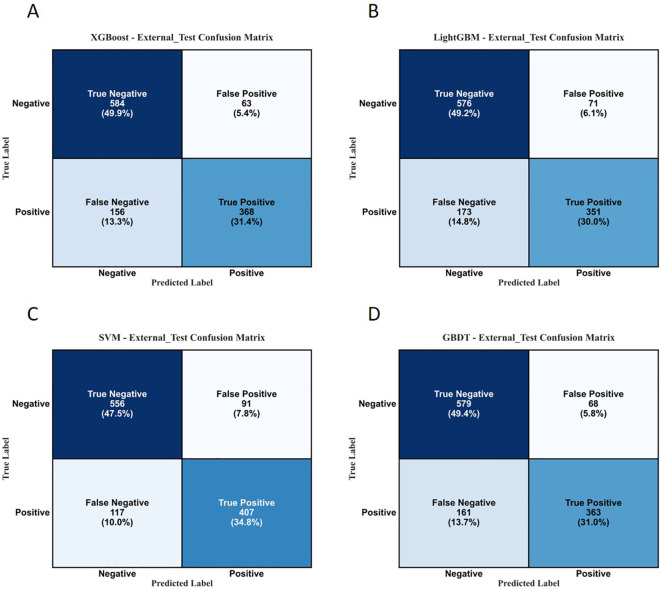
Confusion matrices of each model on the external validation set. **(A)** XGBoost; **(B)** LightGBM; **(C)** SVM; **(D)** GBDT.

The performance evaluation metrics and confusion matrices of each model on the internal test set and external validation set are detailed in **Table 7**; **Figures 14**, **15**.

**Table 7:** Comparison of performance metrics of machine learning models on the internal test set and external validation set.

Dataset	Model	AUC	AP	Accuracy	Precision	Sensitivity	Specificity	F1	Brier
Internal test	XGBoost	0.900	0.871	0.807	0.837	0.702	0.890	0.764	0.132
External test	XGBoost	0.906	0.885	0.813	0.854	0.702	0.903	0.771	0.129
Internal test	LightGBM	0.898	0.873	0.800	0.819	0.707	0.875	0.759	0.134
External test	LightGBM	0.880	0.864	0.792	0.832	0.670	0.890	0.742	0.146
Internal test	SVM	0.898	0.884	0.811	0.821	, 0.737	0.871	0.776	0.129
External test	SVM	0.894	0.873	0.822	0.817	0.777	0.859	0.796	0.133
Internal test	GBDT	0.899	0.884	0.817	0.838	0.732	0.886	0.781	0.129
External test	GBDT	0.885	0.870	0.804	0.842	0.693	0.895	0.760	0.140

**Figure 16:**
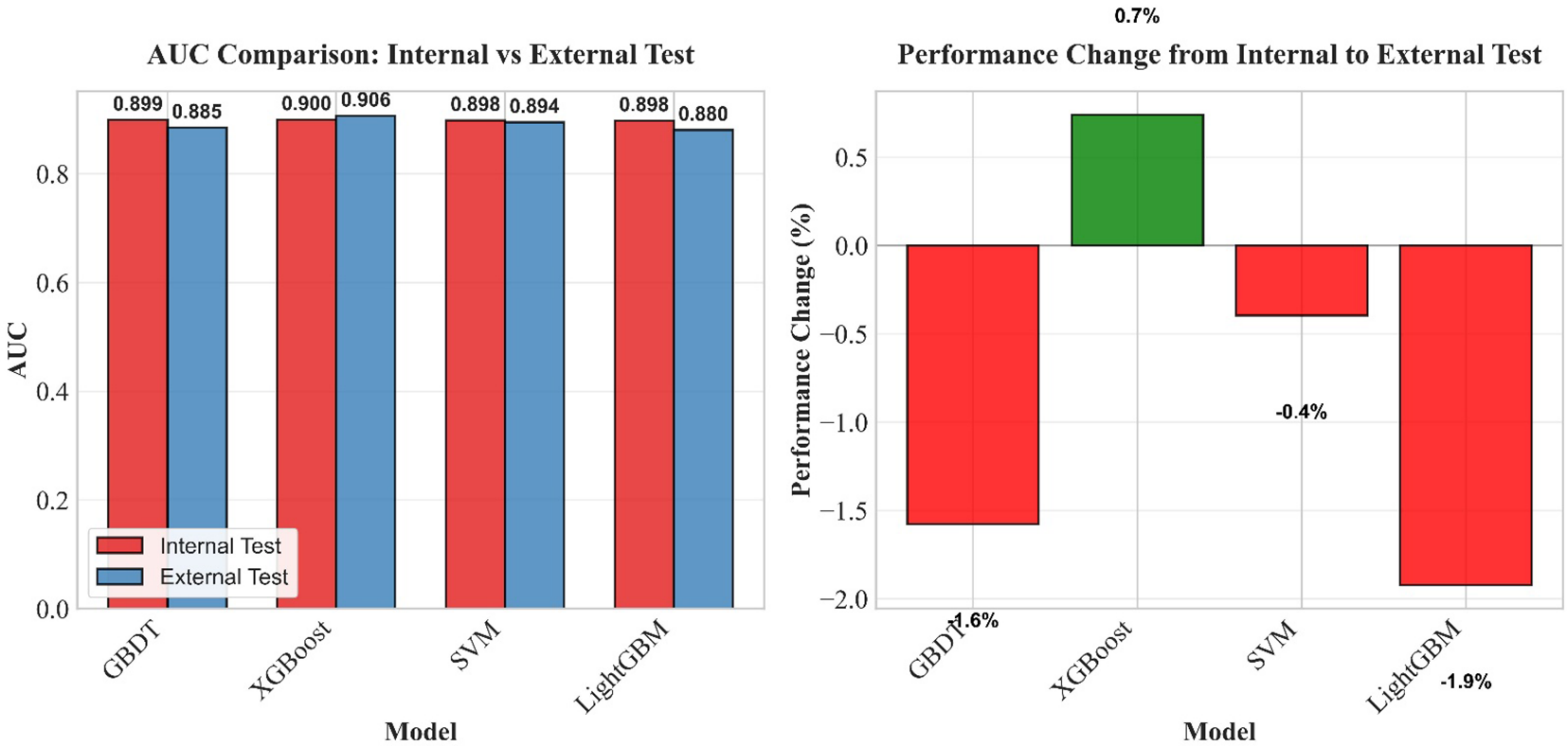
AUC comparison and performance change rates of the four machine learning models on the internal and external test sets.

To more intuitively reflect the differences in generalization stability among different models under the cross−dataset scenario, this study further analyzed the AUC comparison and performance change rates of the models on the internal test set and external validation set, as detailed in **Figure 16** below.

The left panel shows the comparison of AUC values of each model on the internal test set and the external validation set. Red bars represent the internal test set, and blue bars represent the external validation set. The AUC values of all models on the external validation set are slightly lower than those on the internal test set. The right panel shows the AUC performance change rates of each model from the internal test set to the external validation set (negative values indicate performance degradation, and positive values indicate performance improvement). The XGBoost model shows a slight performance improvement (0.7%) on the external validation set, while the other three models exhibit varying degrees of performance degradation, with LightGBM showing the largest decrease (−1.9%). These results intuitively reflect the differences in generalization stability among different models under the cross−dataset scenario.

**Figure 17:**
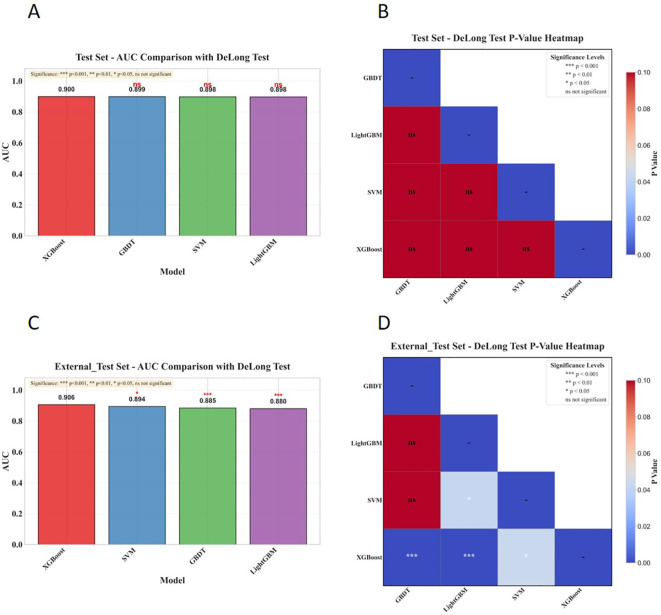
Comparison of AUC performance among machine learning models and results of the DeLong test. **(A)** AUCs of models in the internal test set and annotations of significance from the DeLong test between models; **(B)** Lower-triangle heatmap of P-values from the DeLong test in the internal test set; **(C)** AUCs of models in the external test set and annotations of significance from the DeLong test between models; **(D)** Lower-triangle heatmap of P-values from the DeLong test in the external test set.

The results of the DeLong test for this study were shown in **Figure 17**.

Pairwise comparisons via the DeLong test indicated that, while XGBoost exhibited the highest AUC in the internal test set, no model showed a statistically significant difference in performance relative to others (all *P* ≥ 0.05). In the external validation cohort, by contrast, XGBoost significantly outperformed GBDT and LightGBM (*P* < 0.001 for both) and also achieved a higher AUC than SVM (*P* < 0.05).

### 4.5 Model interpretability analysis results based on SHAP

**Figure 18:**
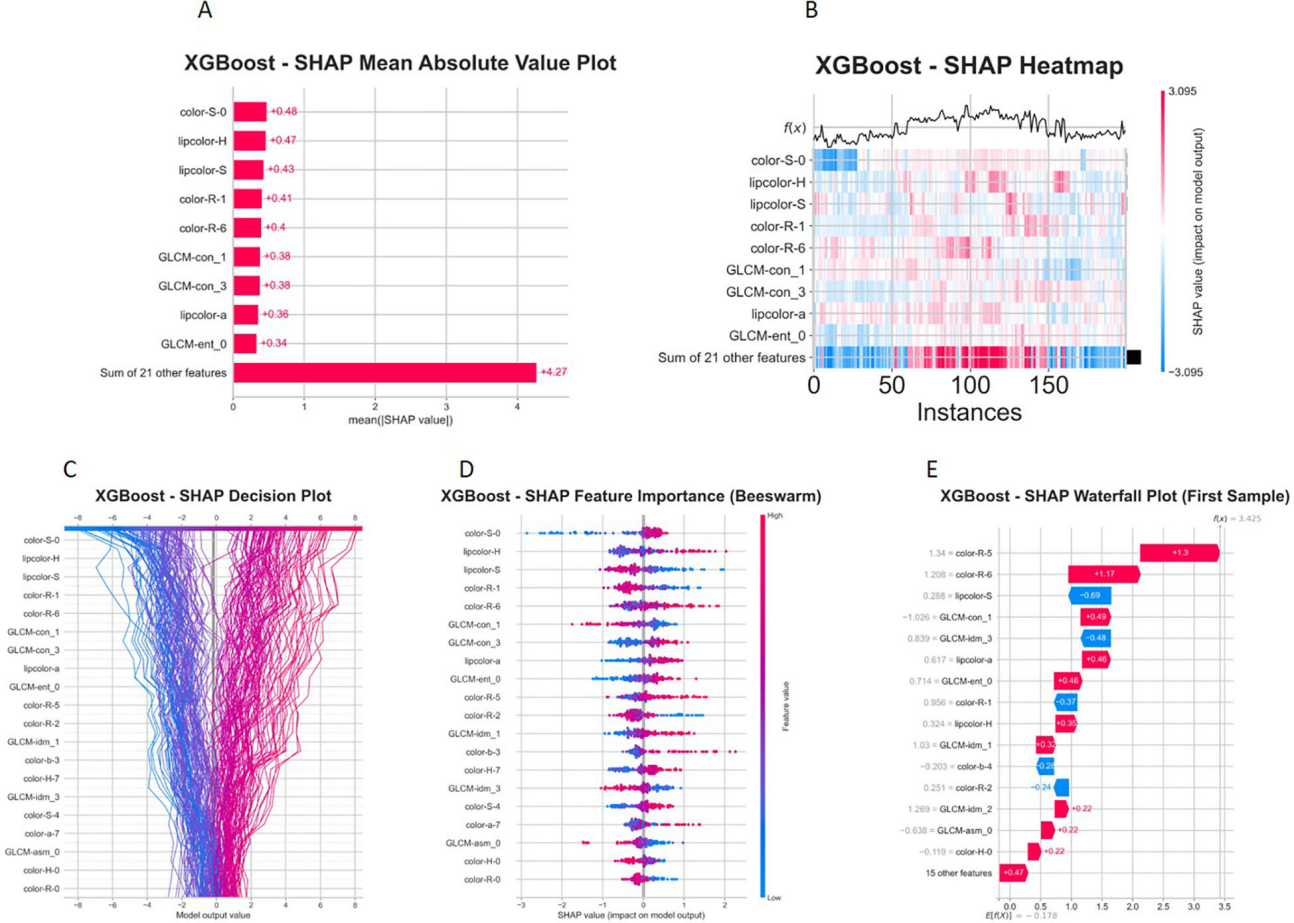
SHAP multidimensional feature explanation visualizations for the XGBoost model. **(A)** SHAP feature importance bar plot. **(B)** Heat map. **(C)** Decision plot. **(D)** Beeswarm summary plot. **(E)** Waterfall plot.

**Figure 19:**
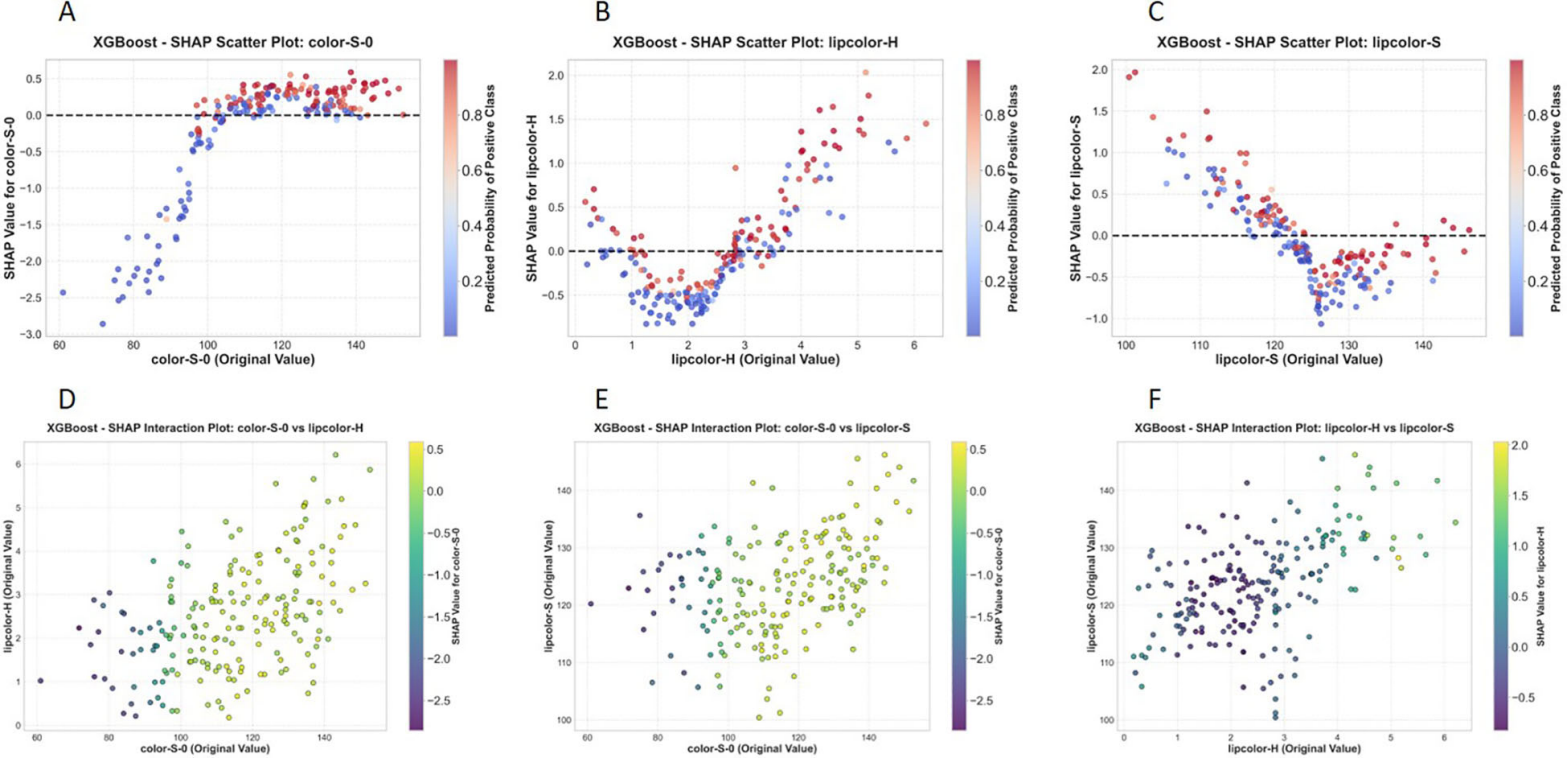
SHAP feature dependency and interaction plots for the XGBoost model. **(A)** SHAP dependence plot for feature color-S-0. **(B)** SHAP dependence plot for feature lipcolor-H. **(C)** SHAP dependence plot for feature lipcolor-S. **(D)** Interaction effect between color-S-0 and lipcolor-H. **(E)** Interaction effect between color-S-0 and lipcolor-S. **(F)** Interaction effect between lipcolor-H and lipcolor-S.

The optimal model (XGBoost) was selected for model interpretability analysis in this study, and the corresponding SHAP analysis results are presented in **Figures 18**, **19** below.

The SHAP feature importance ranking (**Figure 18A**) revealed that color−S−0 made the highest contribution to model prediction (SHAP value ≈ 0.48), followed by lipcolor−H and lipcolor−S, indicating that color space features served as the core decision basis for the XGBoost model. The heatmap (**Figure 18B**) and decision plot (**Figure 17C**) further supplemented the correlation patterns among features and the cumulative contribution process of each feature in individual samples. The beeswarm plot (**Figure 18D**) further uncovered the effect distribution direction of each feature: color−S−0 exhibited an obvious positive driving trend, suggesting that higher color saturation significantly elevated the model output probability; in contrast, lipcolor−H showed a bidirectional distribution, implying a nonlinear threshold effect for lip color hue. The waterfall plot (**Figure 18E**) accurately quantified the stepwise contribution of each feature in a single predicted sample, clarifying the specific effect magnitudes of key driving features and inhibitory features.

Feature dependence analysis (**Figures 19A–C**) demonstrated significant nonlinear associations between the raw values of core features and their SHAP values, indicating that these features exerted an increasing marginal effect on model prediction within moderate value ranges. Feature interaction analysis (**Figures 19D–F**) revealed significant synergistic effects between color−S−0 and lipcolor−H, color−S−0 and lipcolor−S, as well as lipcolor−H and lipcolor−S, illustrating that the model successfully captured the coupling relationships among color features. Such interactions are often masked in single−feature importance assessment but contribute substantially to improving the discriminative ability of the model in actual prediction. These findings not only enhance model transparency but also provide quantitative evidence for the credibility verification of model predictions and subsequent feature optimization.

### 4.6 Modeling results based on baseline-matched populations

To control for potential confounding effects of baseline characteristics such as age and sex on the model, this study further adopted propensity score matching (PSM) to match study participants by sex and age. Finally, 461 participants were included in both the benign pulmonary nodule group and the lung cancer group, respectively. Model training and internal testing were performed using the same analytical approach based on the matched dataset. The results of 10-fold cross-validation of the models on the training set are presented in **Table 8**, and the model performance on the internal test set is summarized in **Table 9**. The ROC curves, PR curves, decision curves, and calibration curves of each model under 10-fold cross-validation in the training set after baseline matching are shown in **Supplementary Figures 2**–**4**, respectively. The performance evaluation curves and confusion matrices of each model on the internal test set are displayed in **Supplementary Figures 5**, **6**.

**Table 8:** Performance evaluation results of models in the training set after baseline matching (10-fold cross-validation).

Model	AUC	AP	Accuracy	Precision	Sensitivity	Specificity	F1
XGBoost	0.818	0.828	0.733	0.749	0.715	0.750	0.729
LightGBM	0.809	0.821	0.727	0.734	0.726	0.728	0.725
SVM	0.815	0.832	0.745	0.761	0.718	0.772	0.737
GBDT	0.822	0.831	0.726	0.739	0.713	0.739	0.723

**Table 9:** Performance evaluation results of models in the internal test set after baseline matching.

Model	AUC	AP	Accuracy	Precision	Sensitivity	Specificity	F1	Brier
XGBoost	0.839	0.849	0.746	0.732	0.772	0.720	0.751	0.167
LightGBM	0.821	0.824	0.724	0.711	0.750	0.699	0.730	0.212
SVM	0.852	0.854	0.795	0.793	0.793	0.796	0.793	0.158
GBDT	0.820	0.815	0.730	0.714	0.761	0.699	0.737	0.175

After baseline matching, all models exhibited favorable discriminative ability in both the training set and the internal test set. As shown in **Table 2**, in the internal test set, the SVM achieved the best overall performance, with an AUC of 0.852, an accuracy of 0.795, an F1-score of 0.793, and the lowest Brier score (0.158), indicating favorable predictive calibration. The XGBoost model also performed prominently in terms of AUC (0.839) and AP (0.849). The remaining models, including LightGBM and GBDT, showed slightly lower performance but still maintained high values across all metrics, suggesting that the constructed models possessed stable and reliable predictive performance on the matched dataset.

## 5 Discussion

### 5.1 Application of facial image analysis technology in the field of medical diagnosis

Big data has strongly driven the in-depth application of artificial intelligence technology in the auxiliary diagnosis of clinical diseases and syndromes, among which facial image analysis technology is gradually becoming an important tool for medical diagnosis. Kosilek RP et al (Kosilek et al., 2015). developed a dedicated facial analysis software for endocrine and hereditary diseases, with a diagnostic sensitivity and specificity both exceeding 80%; Kong X et al (Kong et al., 2024). confirmed that the comprehensive AUC of this technology in the diagnosis of various diseases could reach 0.83 by comprehensively analyzing 42 deep learning-related studies; Kong Y et al (Kong et al., 2020). focused on acromegaly and constructed an automatic diagnosis and severity classification model based on facial photographs, with an AUC of 0.92; Zhang Q et al (Zhang et al., 2021). proposed a dual-stack network (DsNet), which effectively improved the combined diagnostic performance of diabetes and chronic kidney disease through parallel feature extraction and cross-stack attention mechanism. At the methodological level, Thevenot et al (Thevenot et al., 2018). systematically sorted out the technical paths and core challenges of computer vision, providing theoretical support for facial feature extraction and model construction; the deep learning-based facial phenotyping method proposed by Gurovich et al (Gurovich et al., 2019). offered a reference for the optimization of multiple machine learning models; the studies by Hallgrimsson et al (Hallgrímsson et al., 2020). and Choi et al (Choi et al., 2024). in the field of three-dimensional (3D) facial imaging, as well as the relevant achievements in the comparison between two-dimensional (2D) and 3D facial representations (Bannister et al., 2023), all provided empirical evidence for the selection of feature dimensions and representation schemes. These mature technologies and application cases have laid an important foundation for the construction of a lung cancer early warning model based on facial images.

### 5.2 Analysis of image-related studies on lung cancer early warning

Compared with the mature application of facial images in other disease fields, research on lung cancer early warning using facial features is still in its infancy. In contrast, significant progress has been made in lung cancer auxiliary diagnosis via other image modalities: at the histopathological level, researchers proposed deep learning methods based on color features to distinguish lesion types, overcoming the subjective limitations of manual film reading (Bishnoi and Goel, 2023). Other studies use deep learning to analyze biopsy images for EGFR gene mutation prediction, constructing an unannotated model and verifying its generalization performance through multi-center validation (Zhao et al., 2025). In CT imaging, although studies have confirmed that ensemble learning (e.g., the model based on capsule network and visual geometry group (Bushara et al., 2023) and local image feature analysis (Kuo et al., 2021) improve lung cancer diagnosis accuracy, CT examination has the inherent defect of ionizing radiation. Additionally, while the 2D-3D cascaded CNN model enables pulmonary nodule classification, detection and segmentation, its high image quality requirements and computational cost hinder its application in large-scale screening (Dutande et al., 2021). For functional imaging, extracting texture parameters from 68Ga-FAPI-46 PET images effectively quantifies lesion heterogeneity and improves diagnostic accuracy (Wessendorf et al., 2025). These studies provide important technical references for this paper’s model construction. However, existing studies share common limitations: most lack interpretability analysis, restricting their clinical translation (Muhammad and Bendechache, 2024; Rasheed et al., 2022).

Based on the above research foundation, to meet the demand for non-invasive early warning of lung cancer, this study adopted the TFDA-1 tongue diagnosis instrument and integrated multi-color space and texture features to construct a prediction model. With standardized collection, precise imaging, and objective analysis as its core, this instrument has good repeatability, high accuracy, and high compliance, providing reliable scientific and technological support for this study. At present, a number of clinical studies have been carried out based on this instrument (Jiang et al., 2021; Li et al., 2022; Li et al., 2021; Shi et al., 2023). This study systematically explored the application value of facial image features in lung cancer early warning based on large-sample multi-center data of 3470 subjects (2299 cases in the training and development cohort and 1171 cases in the external validation cohort); meanwhile, the SHAP method was used to clarify the core features and their interaction effects, providing support for clinicians to understand the model prediction logic. This study shifts from the traditional, subjective physical examination of TCM to an objective, quantitative analysis of facial color and texture features, using standardized instruments and computer vision algorithms. Through multi-center, large-sample external validation, we ensure robust and reliable results, advancing modern technological support for non-invasive lung cancer early warning based on facial features.

### 5.3 Correlation analysis of facial images

From the perspective of modern biomedical mechanisms, lung cancer, as a systemic disease, can induce systematic pathophysiological changes including chronic inflammation, tissue hypoxia, peripheral microcirculation disturbance, metabolic disorders, and increased oxidative stress. These changes indirectly mediate quantifiable alterations in facial skin related to blood oxygen saturation, pigment distribution, capillary status, and epidermal texture. Our previous study has shown that there were significant differences in facial color space indicators of multiple parts and texture indicators between the benign pulmonary nodule group and the lung cancer group (Shi et al., 2025). The correlation analysis of facial image features in this study also showed that the GLCM texture features and color space features were significantly correlated in both groups. The absolute values of feature correlation coefficients in the benign pulmonary nodule group were generally higher, while the proportion of weak/no correlation features was higher in the lung cancer group, suggesting that the heterogeneity of its feature correlation structure may be stronger. The distinct correlation patterns of facial phenotypic features between benign pulmonary nodules and lung cancer reflect fundamental differences in systemic regulation. This finding not only provides a modern medical evidence-based biological foundation for our model but also establishes a potential non-invasive facial imaging basis for distinguishing malignant from benign lesions. This finding clarifies the core feature association patterns with discriminative value in facial images, lays a data foundation for the subsequent construction of an accurate and efficient pulmonary lesion identification model based on facial image features, and also provides a new research direction for the development of non-invasive pulmonary lesion screening technology.

### 5.4 Model performance evaluation and interpretability analysis

Our previous study has confirmed the potential value of facial color and texture features in lung cancer risk early warning (Shi et al., 2025). To improve the reliability and generalization of the conclusions, this study optimized and expanded on the basis of the previous research. By expanding the sample size, optimizing methodologies, enriching analysis dimensions, and improving the validation system, this study further consolidated the application foundation of facial image features in lung cancer risk early warning, providing more sufficient evidence-based support for the clinical translation of this technology. The four machine learning models (XGBoost, LightGBM, SVM, and GBDT) constructed in this study all showed excellent performance in distinguishing benign and malignant pulmonary lesions, with AUC values maintained above 0.88 in both the internal test set and external validation set, confirming the feasibility and reliability of the modeling approach based on facial image features. Among them, the XGBoost model performed the best, with an AUC of 0.906 in the external validation set, and its performance slightly improved by 0.7% compared with that in the internal test set. Its generalization stability was significantly superior to other models, providing a preferred scheme for clinical translation. The results of the DeLong test revealed an important pattern in model performance: although XGBoost achieved the highest AUC in the internal test set, no statistically significant differences were observed among the four models. However, in the external validation cohort, XGBoost significantly outperformed GBDT, LightGBM, and SVM. This finding indicates that while all models exhibited comparable discriminative ability on single-center data, XGBoost demonstrated superior generalization performance and robustness when tested on data from an independent center. The significant advantage of XGBoost in the external validation set supports our selection of it as the final model, as its stable performance across different populations suggests greater potential for clinical application.

SHAP is a widely adopted model interpretability analysis method, which can effectively address the “black box” problem of machine learning and deep learning models, and significantly improve the clinical reliability and acceptance of such models (Herrero-Tudela et al., 2025). The SHAP interpretability analysis revealed that color-S-0, lipcolor-H, and lipcolor-S were the core decision-making features, and there were significant synergistic interaction effects among these features. This finding not only improved model transparency but also provided quantitative evidence for the research on the correlation mechanism between facial features and pulmonary lesions. These three core features represent facial skin saturation, lip hue, and lip saturation, respectively. These features are mechanistically related to blood oxygen filling, microcirculation status, and pigmentation levels of the skin and lips, and are theoretically consistent with pathological processes such as systemichypoxia, circulatory disturbance, and chronic inflammation to varying degrees. This provides a pathophysiological basis for their application in disease discrimination. Notably, after propensity score matching for age and sex, all models still maintained good discriminative efficacy, with the SVM model achieving an AUC of 0.852, indicating that the predictive value of facial image features for lesions is independent of baseline confounding factors, further enhancing the credibility of the results. In summary, the models constructed in this study have both high discriminability and stable generalization ability, providing an innovative technical path for non-invasive screening of lung cancer. Their core features and interaction patterns also lay a foundation for subsequent model optimization and mechanism exploration.

### 5.5 Limitations

Although this study has achieved some positive results, there are still certain limitations: (1) All the research subjects were from the same region in China and were all Chinese people. There might be certain regional and racial selection biases, and the cross-population generalization ability of the model needs to be further verified. In the future, it is necessary to expand the sample sources and incorporate multi-center, cross-regional, and multi-ethnic data to conduct validation and model optimization. (2) Due to the retrospective data collection conditions of this study, the baseline information of this study lacks traditional lung cancer risk factors such as smoking history and family history. Future prospective cohort studies will further improve the collection and analysis of relevant variables. (3) The model was constructed only based on facial image features, without integrating common clinical indicators such as tumor markers and CT imaging features, which may limit the further improvement of diagnostic efficacy. (4) A cross-sectional design was adopted, which cannot clarify the causal relationship between changes in facial features and the progression of pulmonary lesions. Further verification through longitudinal studies is still needed in the future.

## 6 Conclusion

In summary, based on large-sample multi-center data, this study constructed a machine learning model for lung cancer early warning based on multi-dimensional facial image features. The model has the advantages of non-invasiveness, convenience, strong interpretability, and stable generalization, providing a new idea and potential auxiliary tool for the early screening and identification of lung cancer, and is expected to promote the clinical translation and application of non-invasive diagnostic technology in the field of lung cancer.

## Data Availability

The raw data supporting the conclusions of this article will be made available by the authors, without undue reservation.

## Supplementary material

Supplementary Figure 1 Forest plot of effective facial features.

Supplementary Figure 2 Comparison of ROC curves of the four machine learning models under 10‑fold cross‑validation in the training set based on baseline-matched populations.

Supplementary Figure 3 Comparison of PR curves of the four machine learning models under 10-fold cross-validation in the training set based on baseline-matched populations.

Supplementary Figure 4 Calibration curves and decision curves of the four machine learning models under cross‑validation based on baseline-matched populations. (A) Calibration curves; (B) Decision curves.

Supplementary Figure 5 Multidimensional performance evaluation curves of the four machine learning models on the external test set based on baseline-matched populations. (A) ROC curve; (B) PR curve; (C) Calibration curve; (D) Decision curve.

Supplementary Figure 6 Confusion matrices of each model on the internal test set based on baseline-matched populations. (A) XGBoost; (B) LightGBM; (C) SVM; (D) GBDT.
