# Supplementary figures and images for: Interpretable multi-center machine learning model driven by facial image features for non-invasive early risk assessment of lung cancer
Source: Front Physiol. 2026 May 15;17:1835790. doi: 10.3389/fphys.2026.1835790 (PMC13218986; doi:10.3389/fphys.2026.1835790)

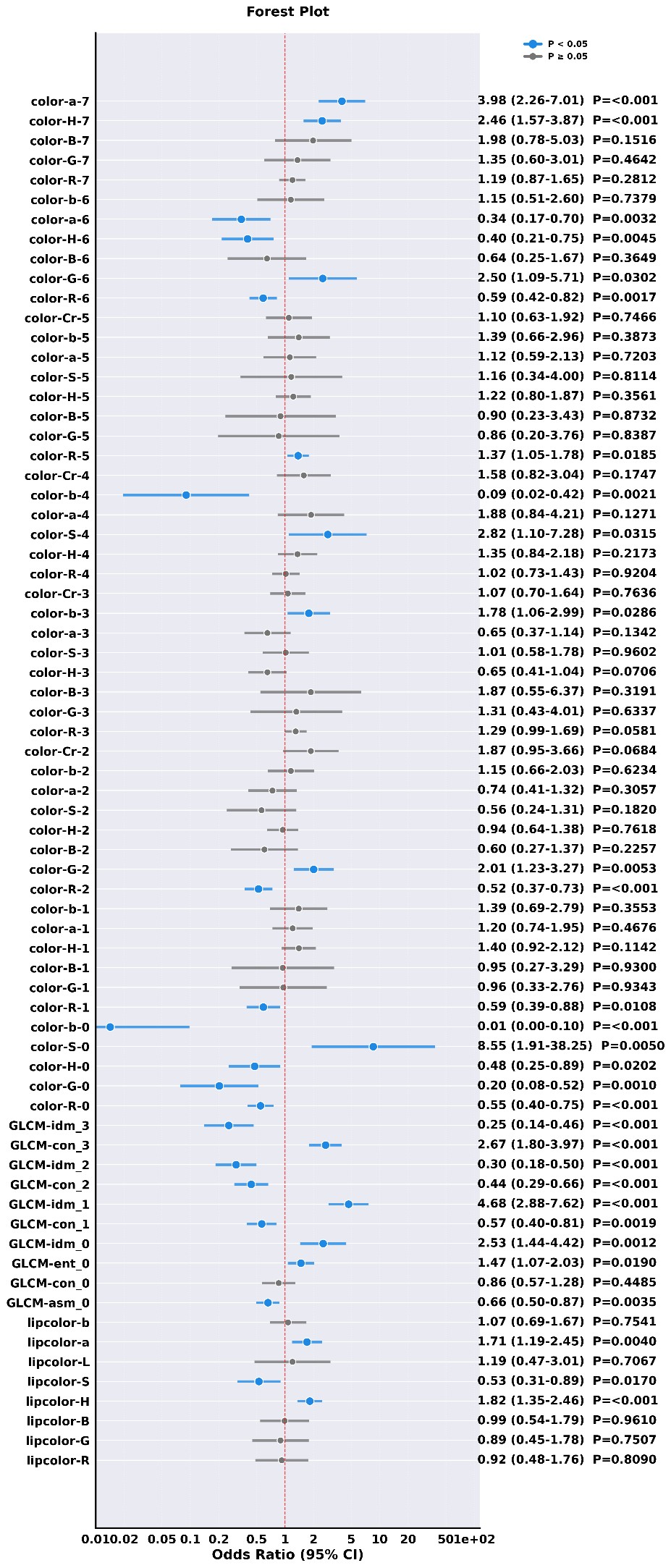

Supplement: Supplementary Figure 1 — Forest plot of effective facial features. [file Image1.jpeg]

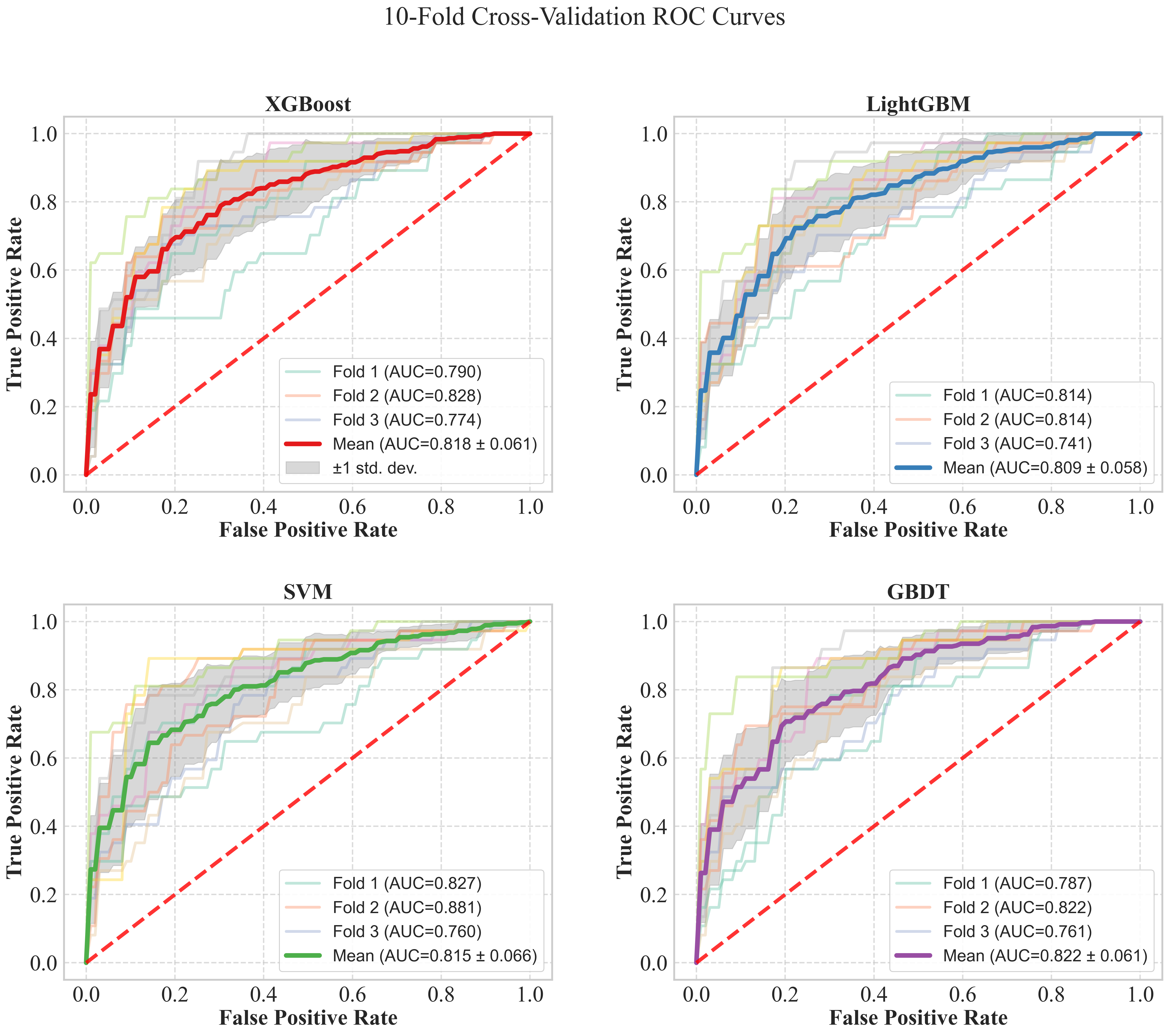

Supplement: Supplementary Figure 2 — Comparison of ROC curves of the four machine learning models under 10‑fold cross‑validation in the training set based on baseline-matched populations. [file Image2.png]

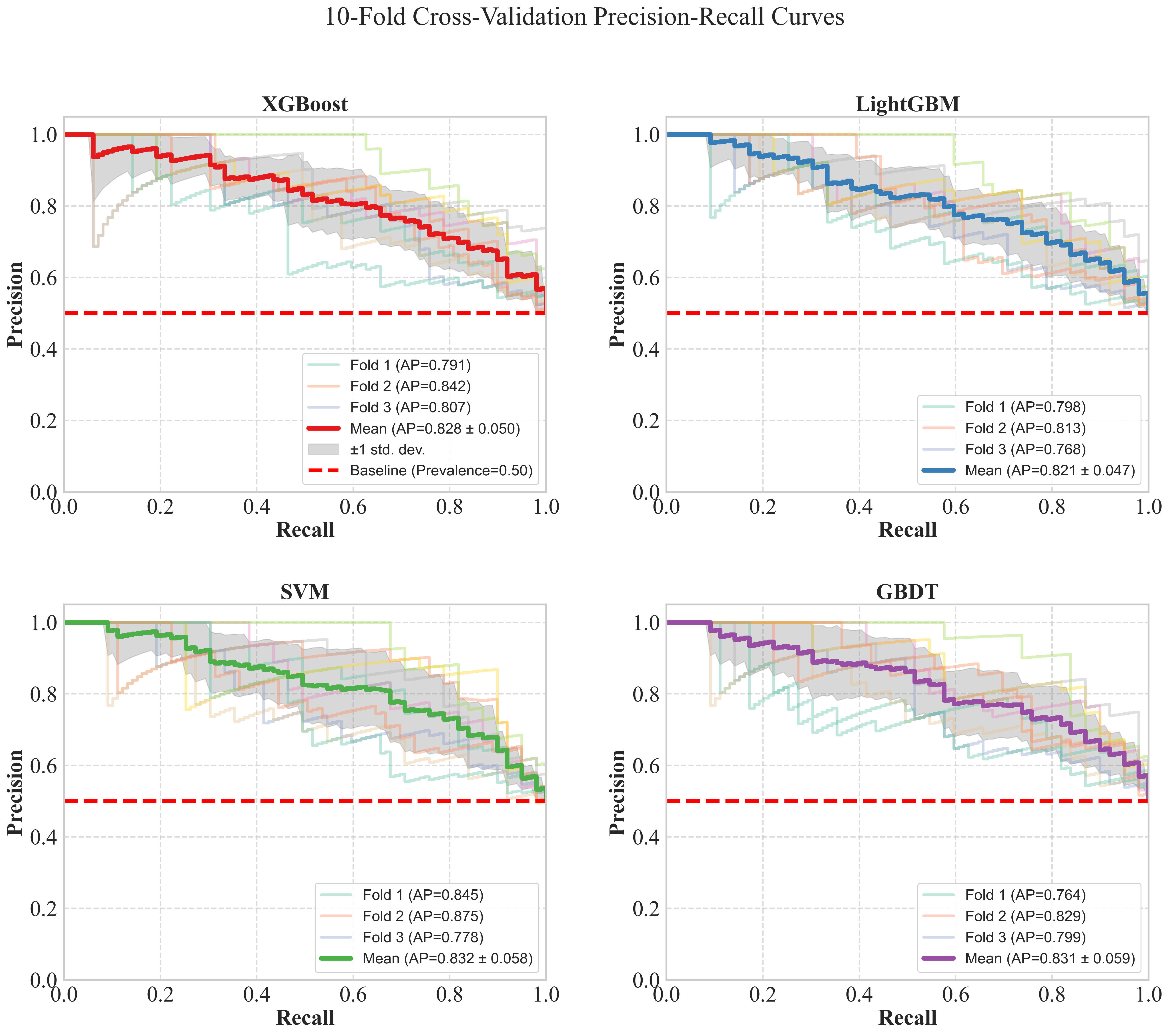

Supplement: Supplementary Figure 3 — Comparison of PR curves of the four machine learning models under 10-fold cross-validation in the training set based on baseline-matched populations. [file Image3.png]

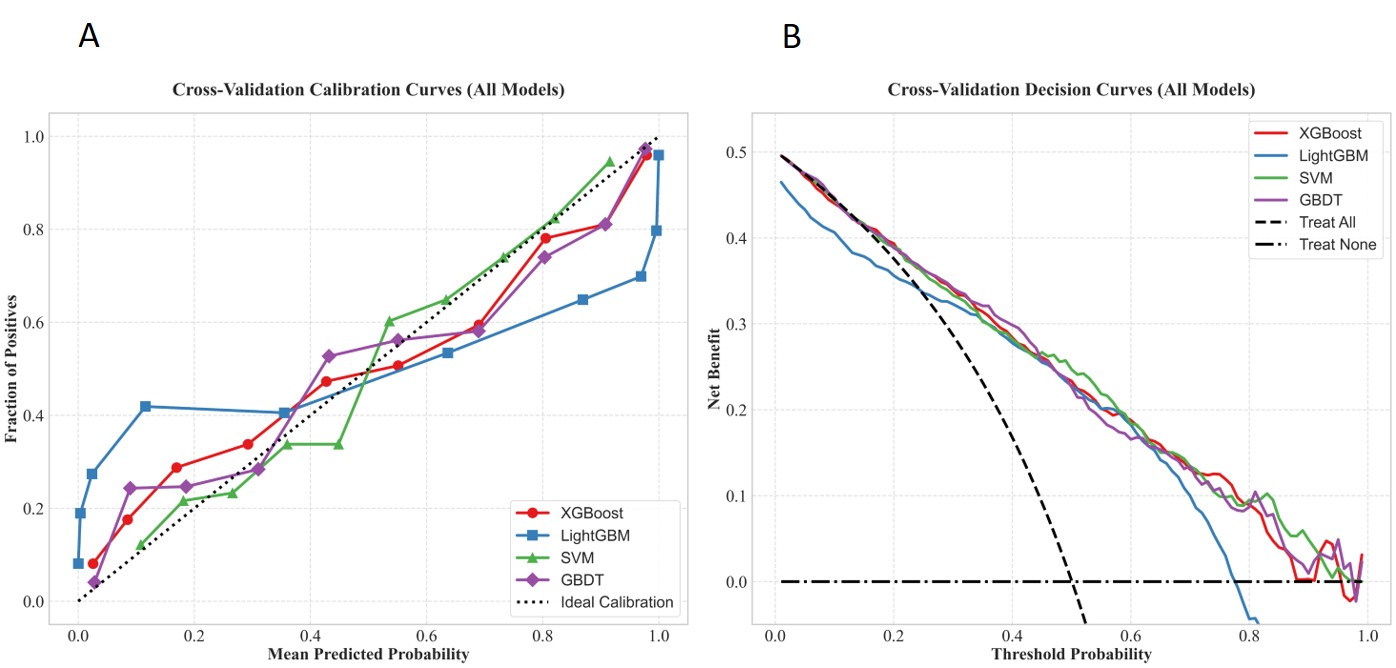

Supplement: Supplementary Figure 4 — Calibration curves and decision curves of the four machine learning models under cross‑validation based on baseline-matched populations. (A) Calibration curves; (B) Decision curves. [file Image4.jpeg]

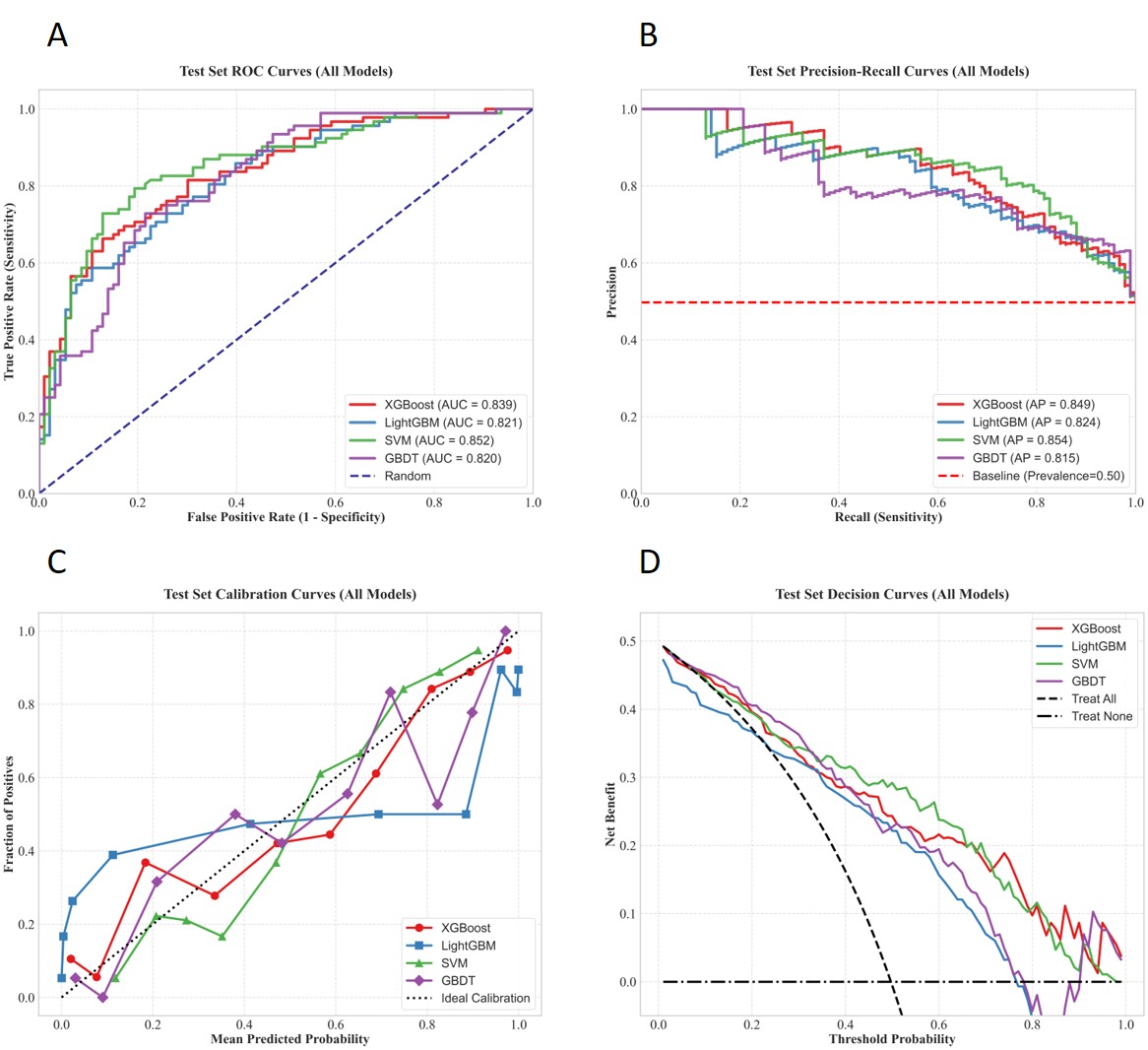

Supplement: Supplementary Figure 5 — Multidimensional performance evaluation curves of the four machine learning models on the external test set based on baseline-matched populations. (A) ROC curve; (B) PR curve; (C) Calibration curve; (D) Decision curve. [file Image5.jpeg]

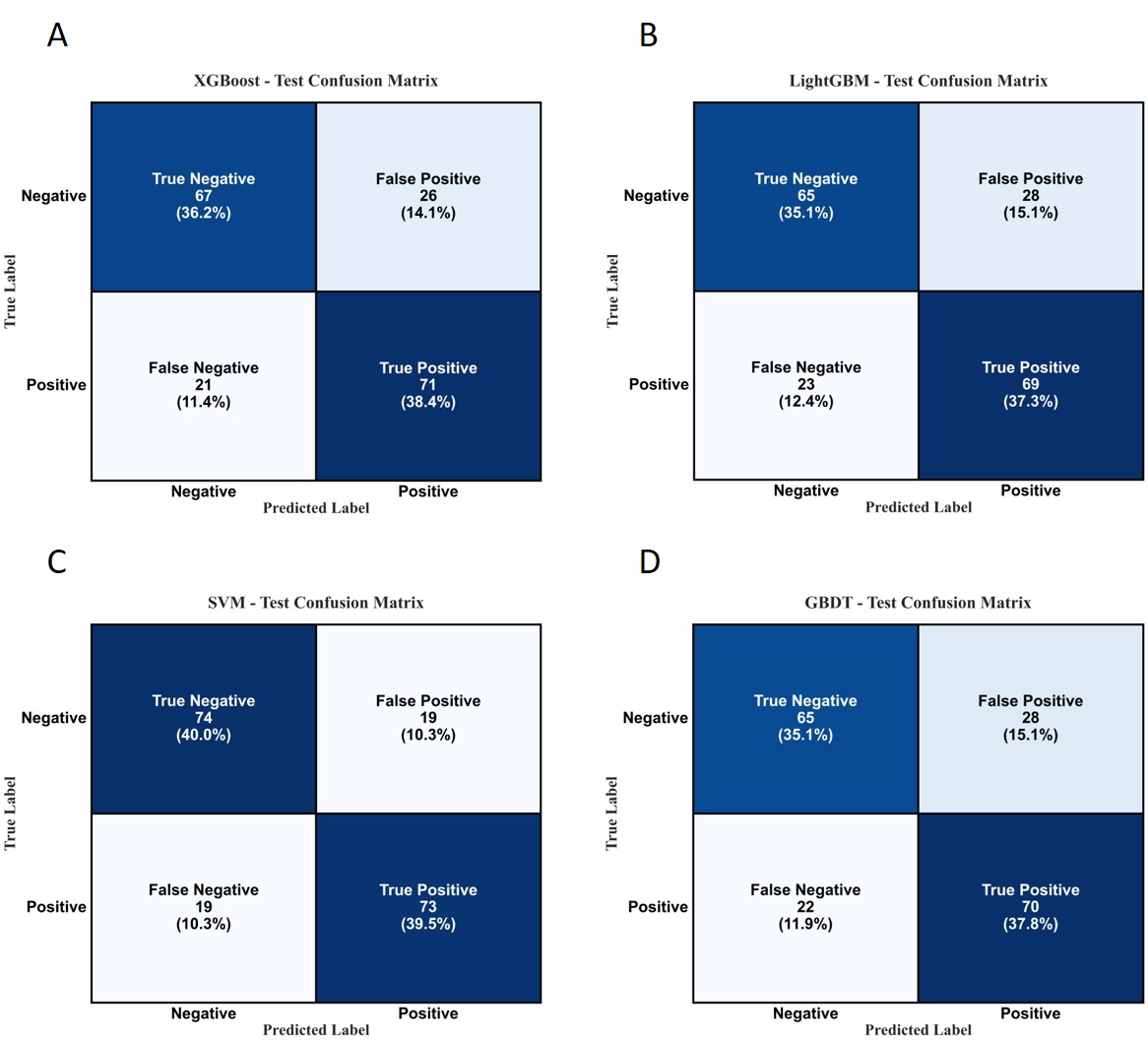

Supplement: Supplementary Figure 6 — Confusion matrices of each model on the internal test set based on baseline-matched populations. (A) XGBoost; (B) LightGBM; (C) SVM; (D) GBDT. [file Image6.jpeg]
