# Supplementary material for: Interpretable multi-center machine learning model driven by facial image features for non-invasive early risk assessment of lung cancer
Source: Front Physiol. 2026 May 15;17:1835790. doi: 10.3389/fphys.2026.1835790 (PMC13218986; doi:10.3389/fphys.2026.1835790)
Supplement: Supplementary file 7 [file Table1.docx]

Supplementary Table S1. The ten-fold cross-validation model results of each model based on the training set

| Model | Fold | AUC | AP | Accuracy | Precision | Sensitivity | Specificity | F1 |
| --- | --- | --- | --- | --- | --- | --- | --- | --- |
| GBDT | 1 | 0.787 | 0.764 | 0.716 | 0.682 | 0.811 | 0.622 | 0.741 |
| GBDT | 2 | 0.822 | 0.829 | 0.703 | 0.674 | 0.784 | 0.622 | 0.725 |
| GBDT | 3 | 0.761 | 0.799 | 0.649 | 0.641 | 0.676 | 0.622 | 0.658 |
| GBDT | 4 | 0.856 | 0.871 | 0.716 | 0.735 | 0.676 | 0.757 | 0.704 |
| GBDT | 5 | 0.914 | 0.931 | 0.838 | 0.838 | 0.838 | 0.838 | 0.838 |
| GBDT | 6 | 0.876 | 0.882 | 0.797 | 0.824 | 0.757 | 0.838 | 0.789 |
| GBDT | 7 | 0.764 | 0.768 | 0.676 | 0.710 | 0.595 | 0.757 | 0.647 |
| GBDT | 8 | 0.881 | 0.865 | 0.753 | 0.806 | 0.676 | 0.833 | 0.735 |
| GBDT | 9 | 0.712 | 0.736 | 0.616 | 0.622 | 0.622 | 0.611 | 0.622 |
| GBDT | 10 | 0.843 | 0.863 | 0.795 | 0.862 | 0.694 | 0.892 | 0.769 |
| LightGBM | 1 | 0.814 | 0.798 | 0.743 | 0.705 | 0.838 | 0.649 | 0.765 |
| LightGBM | 2 | 0.814 | 0.813 | 0.743 | 0.705 | 0.838 | 0.649 | 0.765 |
| LightGBM | 3 | 0.741 | 0.768 | 0.635 | 0.619 | 0.703 | 0.568 | 0.658 |
| LightGBM | 4 | 0.857 | 0.857 | 0.824 | 0.833 | 0.811 | 0.838 | 0.822 |
| LightGBM | 5 | 0.897 | 0.915 | 0.784 | 0.756 | 0.838 | 0.730 | 0.795 |
| LightGBM | 6 | 0.828 | 0.835 | 0.770 | 0.794 | 0.730 | 0.811 | 0.761 |
| LightGBM | 7 | 0.774 | 0.790 | 0.716 | 0.750 | 0.649 | 0.784 | 0.696 |
| LightGBM | 8 | 0.883 | 0.870 | 0.699 | 0.759 | 0.595 | 0.806 | 0.667 |
| LightGBM | 9 | 0.703 | 0.753 | 0.644 | 0.641 | 0.676 | 0.611 | 0.658 |
| LightGBM | 10 | 0.779 | 0.815 | 0.712 | 0.778 | 0.583 | 0.838 | 0.667 |
| SVM | 1 | 0.827 | 0.845 | 0.757 | 0.744 | 0.784 | 0.730 | 0.763 |
| SVM | 2 | 0.881 | 0.875 | 0.811 | 0.811 | 0.811 | 0.811 | 0.811 |
| SVM | 3 | 0.760 | 0.778 | 0.689 | 0.694 | 0.676 | 0.703 | 0.685 |
| SVM | 4 | 0.831 | 0.853 | 0.743 | 0.781 | 0.676 | 0.811 | 0.725 |
| SVM | 5 | 0.907 | 0.927 | 0.784 | 0.769 | 0.811 | 0.757 | 0.789 |
| SVM | 6 | 0.866 | 0.863 | 0.824 | 0.853 | 0.784 | 0.865 | 0.817 |
| SVM | 7 | 0.718 | 0.731 | 0.703 | 0.714 | 0.676 | 0.730 | 0.694 |
| SVM | 8 | 0.865 | 0.884 | 0.781 | 0.839 | 0.703 | 0.861 | 0.765 |
| SVM | 9 | 0.703 | 0.762 | 0.644 | 0.649 | 0.649 | 0.639 | 0.649 |
| SVM | 10 | 0.789 | 0.798 | 0.712 | 0.759 | 0.611 | 0.811 | 0.677 |
| XGBoost | 1 | 0.790 | 0.791 | 0.703 | 0.692 | 0.730 | 0.676 | 0.711 |
| XGBoost | 2 | 0.828 | 0.842 | 0.757 | 0.732 | 0.811 | 0.703 | 0.769 |
| XGBoost | 3 | 0.774 | 0.807 | 0.716 | 0.711 | 0.730 | 0.703 | 0.720 |
| XGBoost | 4 | 0.861 | 0.860 | 0.757 | 0.788 | 0.703 | 0.811 | 0.743 |
| XGBoost | 5 | 0.914 | 0.928 | 0.824 | 0.816 | 0.838 | 0.811 | 0.827 |
| XGBoost | 6 | 0.859 | 0.856 | 0.784 | 0.818 | 0.730 | 0.838 | 0.771 |
| XGBoost | 7 | 0.767 | 0.773 | 0.689 | 0.694 | 0.676 | 0.703 | 0.685 |
| XGBoost | 8 | 0.879 | 0.847 | 0.753 | 0.806 | 0.676 | 0.833 | 0.735 |
| XGBoost | 9 | 0.696 | 0.738 | 0.589 | 0.585 | 0.649 | 0.528 | 0.615 |
| XGBoost | 10 | 0.812 | 0.835 | 0.753 | 0.846 | 0.611 | 0.892 | 0.710 |
